# State of the Art and Consensus Statements by Healthcare Providers, Patients, and Caregivers on Continuous Glucose Monitoring in Liver Glycogen Storage Diseases

**DOI:** 10.1002/jimd.70040

**Published:** 2025-05-13

**Authors:** Terry G. J. Derks, Ruben J. Overduin, Sarah C. Grünert, Alessandro Rossi, CGM GSD Collaborators Group, Terry G. J. Derks, Ruben J. Overduin, Bibiana M. de Oliveira, Julien H. Park, David Weinstein, Diva D. De Leon, Yuval E. Landau, Alessandro La Rosa, Damian Cohen, Anke Schumann, Anne Kwok Mei Kwun, Yunkoo Kang, Foekje de Boer, María Clemente, Wendy E. Smith, Margo Sheck Breilyn, Rebecca Riba‐Wolman, Karen Loechner, Malaya Mount, John Mitchell, Samantha Vergano, Stefanie Rosenbaum‐Fabian, Shaylee King, Iris Ferrecchia, Kaustuv Bhattacharya, David F. Rodriguez‐Buritica, Katalin Ross, Anastasia Skouma, Allan M. Lund, Elizabeth Robinson, Caitie Hessenthaler, Emma Corcoran, Tassia Tonon, Daniela Karall, Laura Sliwoski, Ulrike Steuerwald, Patricia Pérez Mohand, Julieta Bonvin Sallago, Nerea López Maldonado, Renee Bethel, Monica Boyer, Timothy Fazio, Vincenza Gragnaniello, María‐Luz Couce, Chiara Falanga, Urh Groselj, Brandy Rawls‐Castillo, Zazil Olivares‐Sandoval, Carolina F. Moura de Souza, Sabine Scholl‐Buergi, Annalisa Madeo, Julia B. Hennermann, Eva Venegas, Arianna Maiorana, Dorothea Haas, Benjamin Weil, Eva Thimm, Alboren Shtylla, Thomas Opladen, Thomas Scherer, Danielle K. Bourque, Tergita Preci, Michel Hochuli, Thomas Casswall, Ellen Crushell, Shagun Kaur, Petra Zsidegh, Risto Lapatto, Matthias Gautschi, Dorothea Möslinger, Peter Witters, Ute Stachelhaus‐Theimer, Flannery Tomberlin, Ana Drole Torkar, Elena Procopio, Luisa Diogo Matos, Saskia Wortmann, Julia Neugebauer, Teresa Rink, Natalie Weinhold, Suresh Vijay, Krista Engen, Jean‐Marc Nuoffer, Andrea Haijer‐Schreuder, Peter Freisinger, Monika Williams, Patricia Janeiro, Justė Parnarauskienė, Çiğdem Seher Kasapkara, Miguel Angel Martinez Olmos, Areeg El‐Gharbawy, Heather Saavedra, Nicola Longo, Magali Reyes Apodaca, Sunita Bijarnia‐Mahay, Leyla Tümer, Martina Huemer, René Santer, Lina Ghaloul‐Gonzalez, Katherine Anderson, Rebecca L. Koch, Christian Kogelmann, Annamaria Sapuppo, Brian Shayota, Melanie M. van der Klauw, Fatih Ezgu, Clemens Kamrath, Camilla Carøe, Karolina M. Stepien, Fiona White, Sarah C. Grünert, Alessandro Rossi

**Affiliations:** ^1^ Department of Metabolic Diseases Beatrix Children's Hospital, University Medical Center Groningen, University of Groningen Groningen the Netherlands; ^2^ UMCG Center of Expertise for Carbohydrate, Fatty Acid Oxidation and Ketone Bodies Disorders University Medical Center Groningen Groningen the Netherlands; ^3^ Department of General Pediatrics, Adolescent Medicine and Neonatology, Faculty of Medicine Medical Center‐University of Freiburg Freiburg Germany; ^4^ Department of Translational Medicine, Section of Pediatrics University of Naples “Federico II” Naples Italy

**Keywords:** glycemic control, management, rare disease, survey

## Abstract

Continuous glucose monitoring (CGM) is increasingly used although not officially registered for the management of people living with liver glycogen storage diseases (GSDs). The aims of this study were twofold: (a) to investigate the current experiences of healthcare providers (HCPs), patients, and caregivers using CGM to monitor glucose concentrations in liver GSDs, and (b) to formulate consensus statements. Two web‐based questionnaires were distributed, one for HCPs and one for patients and/or their caregivers. The questionnaires collected data on demographics and epidemiology, current use of CGM, and opinions and statements about CGM in GSDs. For the statements, respondents rated their agreement on a 5‐point Likert scale, and the consensus level was set at 75%. One Hundred Fourteen HCPs (including 87 physicians and 26 dietitians) from 28 countries responded, representing care of approximately 3800 liver GSD patients. Additionally, 148 GSD patients and/or their caregivers from 21 countries responded, mainly representing GSD Ia (*n* = 50), GSD Ib (*n* = 56), GSD III (*n* = 14), and GSD IX (*n* = 18). The median age to consider starting to use CGM was 6 and 2 months for HCPs and GSD families, respectively. Out of 16 statements common to the two questionnaires, HCPs and patients/caregivers reached consensus on 12 statements in both groups. Use of CGM is considered standard of care by both HCPs and GSD families, but reimbursement of CGM devices is challenging. Compared to diabetes mellitus, CGM should be applied differently in liver GSDs. Consensus guidelines are warranted on the use of CGM in liver GSDs, both in routine healthcare and in clinical trials.

**Abbreviations**
CGM: continuous glucose monitoring
DM: diabetes mellitus
FBS: Fanconi‐Bickel syndrome
GSD: glycogen storage disease
HCP: healthcare provider
SMBG: self‐monitoring blood glucose
TAR: time above range
TBR: time below range
TIR: time in range

## 1 Introduction

Liver glycogen storage diseases (GSD) are a group of rare, inherited disorders of carbohydrate metabolism characterized by a defect in the synthesis or breakdown of glycogen in the liver [1]. Liver GSDs include GSD subtypes 0a, I, III, IV, VI, IX, and XI (also called Fanconi‐Bickel syndrome; FBS). Fasting hypoglycemia is a common symptom in liver GSDs. Hence, monitoring blood glucose concentrations is paramount in the management of people with liver GSDs. For decades, self‐monitoring by using blood glucose (SMBG) meters and measuring finger stick capillary blood glucose concentration has been the traditional method for assessing glycemic control and confirming hypoglycemia at home. These SMBG meters and the finger stick techniques were originally developed for and adopted from the care of patients with diabetes mellitus (DM).

Continuous glucose monitoring (CGM) systems are medical devices that measure glucose in interstitial tissue fluid and are currently officially registered for use in people with DM. Official approvals by national authorities and insurance companies vary from country to country. For example, the FDA has approved the Dexcom G6/G7 for use in people with DM from the age of two and the Freestyle Libre 2/3 from the age of four. After the introduction of CGM systems, CGM‐based glycemic targets (e.g., time in range (TIR), time below range (TBR), time above range (TAR), amongst others) have been defined to guide clinical decisions in DM, both in routine healthcare [2, 3] and clinical trials [4]. In the more recent years, the accuracy and precision of CGM devices, particularly in the low blood glucose ranges, have been gradually improved [5, 6, 7]. Consequently, CGM devices have been used for diagnosis and follow‐up in other disorders of glucose homeostasis that are associated with hypoglycemias, including but not limited to post‐bariatric hypoglycemia [8], congenital hyperinsulinism [9], and liver GSDs [10, 11, 12]. Despite its added value to detect and monitor unrecognized changes of glucose concentrations, published data on CGM metrics in liver GSDs are scarce due to a vicious cycle of the rarity of these disorders, inconsistent use for various reasons, reimbursement challenges, and legal issues. Recently, the changes from baseline percentages of glucose levels in TBR < 70 mg/dL (< 3.9 mmol/L) and in TIR 70–120 mg/dL (3.9–6.7 mmol/L) have been included as secondary outcome parameters in GSD Ia gene transfer clinical trials (such as NCT05139316) underlining the growing importance of CGM in GSDs.

The two aims of this study were (1) to investigate the current experiences of GSD healthcare providers (HCPs), patients, and caregivers using CGM, and (2) to formulate consensus statements on the use of CGM in liver GSDs. The authors consider this study as the first step towards consensus guidelines, both for routine healthcare settings of GSD patient management and clinical trials.

## 2 Methods

### 2.1 Ethics

For this non‐interventional questionnaire study collecting anonymized data, no ethics approval was necessary.

### 2.2 Questionnaires

TGJD, SCG, RO, and AR designed two SurveyMonkey web‐based questionnaires: Questionnaire 1 (Q1) was developed for HCPs, and Questionnaire 2 (Q2) was developed for people with a liver GSD and/or caregivers. Both questionnaires are available as Supporting Information (Files S1 and S2). Briefly, Q1 and Q2 consisted of 49 and 34 questions respectively and had 31 questions in common. Both questionnaires collected information in the following three categories: (1) demographic and background information (and contact details as well as details on followed patients for HCPs), (2) current use of CGM, and (3) opinions and statements about CGM use in GSDs. The invitation to fill out the questionnaires was distributed on February 04, 2024, with a reminder sent on March 05, 2024. Data collection closed on March 18, 2024.

The link to Q1 was distributed via e‐mail to HCPs taking care of people with liver GSDs who: (a) have been corresponding authors in previous publications about this topic; (b) collaborate within MetabERN (invitations sent directly and via the MetabERN coordination office); (c) collaborate within GSD Value (i.e., an international collaborative project to establish a person‐centered standard outcome set for liver GSD); (d) have participated in the International Priority Setting Partnership for liver GSDs [13]; (e) work in centers where clinical trials in liver GSDs are conducted (information retrieved via ClinicalTrials.gov on December 29, 2023); (f) are members of Genetic Metabolic Dietitians International (GMDI), and/or (g) are active on the Metab‐L listserv (https://www.daneel.franken.de/metab‐l/).

The link to Q2 was distributed via e‐mail to the following patient organizations: Associação Brasileira de Glicogenose (ABGLICO, Brazil), Association Francophone des Glycogénoses (AFG), Association for Glycogen Storage Disease (AGSD, USA), Association for Glycogen Storage Disease–UK (AGSD‐UK), Associazione Italiana Glicogenosi (AIGlico), Asociacion Española de Enfermos de Glucogenosis (A.E.E.G), Belgische Organisatie voor Kinderen en volwassenen met een Stofwisselingsziekte vzw (BOKS), Canadian Association for Glycogen Storage Disease, Fuglucol Fundacion Glucogenosis de Colombia, Selbsthilfegruppe Glykogenose Deutschland e.V. (SHG Glykogenose, Germany), Glucolatino (Latin America), Scandinavian Association for Glycogen Storage Disease (SAGSD) and Volwassen Kinderen en Stofwisselingsziekten (VKS, The Netherlands).

The results were discussed in a final workshop during the Annual Symposium of the Society for the Study of Inborn Errors of Metabolism (SSIEM) in Porto, Portugal on September 02, 2024. All respondents to Q1 were invited to participate either in person or virtually. In preparation for the workshop, the respondents received a preliminary draft of the manuscript (which included Table 1) and summaries of the answers to all questions of Q1 and Q2. Preliminary data from this project were also presented as a poster at the SSIEM symposium in Porto, Portugal on September 03–06, 2024.

**TABLE 1:** Consensus on statements regarding the use of CGM amongst HCPs (Q1; *n* = 114) and GSD patients and caregivers (Q2; *n* = 148).

Question numbers in Q1/Q2	Statements	HCPs	GSD families
29/16	Liver GSD patients should be able to use CGM as a standard of care.	86.8% (99/114)	89.8% (132/147)
30/17	Liver GSD patients should be able to use unblinded CGM (i.e., families can immediately see the glucose values).	75.4% (86/114)	84.9% (124/146)
31/18	Real‐time CGM in liver GSD patients has additional value for patients.	90.3% (102/113)	93.2% (136/146)
32/19	Real‐time CGM in liver GSD patients has additional value for their caregivers.	92.9% (105/113)	95.1% (136/143)
33/20	Real‐time CGM in liver GSD patients has additional value for healthcare providers.	88.5% (100/113)	82.1% (119/145)
34/21	The advantages of CGM in patients with liver GSD outweigh the disadvantages.	88.5% (100/113)	87.6% (127/145)
35/22	Ideal CGMvalues should meet the needs of an individual patient.	86.9% (99/114)	87.5% (126/144)
36/23	General recommendations (i.e., guidelines) on ideal CGM‐values should be defined for patients with liver GSD.	92.9% (105/113)	81.4% (118/145)
37/24	CGM‐based glycemic targets depend on the liver GSD subtype.	67.3% (76/113)	68.8% (99/144)
38/25	CGM sensor glucose data should also be reported separately for nocturnal (00:00–05:59 h) and daytime periods (06:00–23:59 h).	53.6% (60/112)	59.0% (85/144)
39/26	Integrating information on physical activity is paramount to adequately interpret CGM metrics in patients with liver GSD.	96.5% (110/114)	79.0% (113/143)
40/27	Integrating information on the actual diet is paramount to adequately interpret CGM metrics in patients with liver GSD.	96.5% (110/114)	78.3% (112/143)
41/28	Level 1 hypoglycemia glucose level of 54–69 mg/dL (3.0–3.9 mmol/L) assessed by CGM, should be considered an alert threshold independent of any acute symptoms.	71.1% (81/114)	76.9% (110/143)
42/29	Level 2 hypoglycemia glucose level of less than 54 mg/dL (< 3.0 mmol/L) assessed by CGM, with or without symptoms, should be considered clinically significant for a liver GSD patient and requires immediate attention.	92.1% (105/114)	84.0% (121/144)
43/30	In patients with liver GSD, the first priority is to reduce time‐below‐range (TBR) to target levels and then address time in range (TIR) or time‐above‐range (TAR) targets.	82.5% (94/114)	79.3% (111/140)
46/31	It is important to assess hyperglycemia after meals or percentage points of time‐above‐range.	86.8% (99/114)	70.8% (102/144)

*Note:* The column entitled “questions” contains the numbers of the questions in Q1 and Q2, for the HCPs and GSD families respectively. Statements with consensus within the group of HCPs or GSD families are in green; no consensus is depicted in red.

### 2.3 Definitions and Data Analysis

All data are presented with descriptive statistics. Results are expressed as percentages and medians (ranges); results in figures are expressed as percentages. For the statements in Q1 and Q2, respondents rated their agreement on a 5‐point Likert scale. Consensus on a given statement was considered reached when 75% or more of the respondents answered “agreed” or “strongly agreed.”

## 3 Results

### 3.1 General Characteristics of the Respondents

**FIGURE 1:**
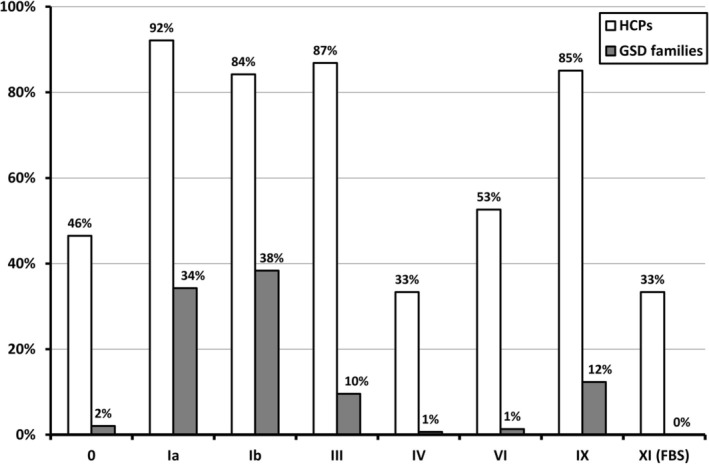
Liver GSD subtypes managed by HCPs within their hospitals (Q1, question 11, open columns) and type of liver GSD in participating patients and caregivers (Q2, question 4; grey columns). The *X*‐axis displays the different liver GSD subtypes. The *Y*‐axis displays either the percentages of HCPs managing patients with the respective GSD subtype (in white bars), or the percentage distribution of GSD subtypes among all GSD patients and caregivers who responded to Q2 (grey bars).

Q1 was answered by 114 HCPs originating from 28 countries (File S3), taking care of approximately 3800 patients with liver GSDs. For several questions, there were multiple answer options, which explain why the presented summed values and percentages may exceed the number of respondents or 100%, respectively. Most HCPs were physicians (*n* = 87), followed by dietitians (*n* = 26) and researchers (*n* = 17). Among these HCPs, four were also individuals with a liver GSD, and three were caregivers of a person with liver GSD. Most of the HCPs took care of both children and adults (75/114; 66%), whilst 42% (48/114) were experienced in using CGM in patients with DM. Of the total of 114 HCPs, the following number (in brackets) indicated that patients with the following diagnoses were cared for in person or at the respective facility: GSD 0 (*n* = 53), GSD Ia (*n* = 105), GSD Ib (*n* = 96), GSD III (*n* = 99), GSD IV (*n* = 38), GSD VI (*n* = 60), GSD IX (*n* = 97), and FBS (*n* = 38). Figure 1 presents the percentages of HCPs who indicated that they manage patients with these specific GSD subtypes in their hospitals. This figure also shows the percentages of GSD subtypes among all Q2 respondents. Q2 was answered by 148 liver GSD patients and/or their caregivers, originating from 21 countries (File S3). The Q2 respondents mainly represented GSD Ia (*n* = 50), GSD Ib (*n* = 56), GSD III (*n* = 14), and GSD IX (*n* = 18). Most of these respondents were caregivers (*n* = 97), followed by individuals with GSD (*n* = 51), and some belonging to the group of GSD families were also HCPs (*n* = 3).

### 3.2 Utilization of CGM in Liver GSD by Healthcare Providers, Patients, and Caregivers

**FIGURE 2:**
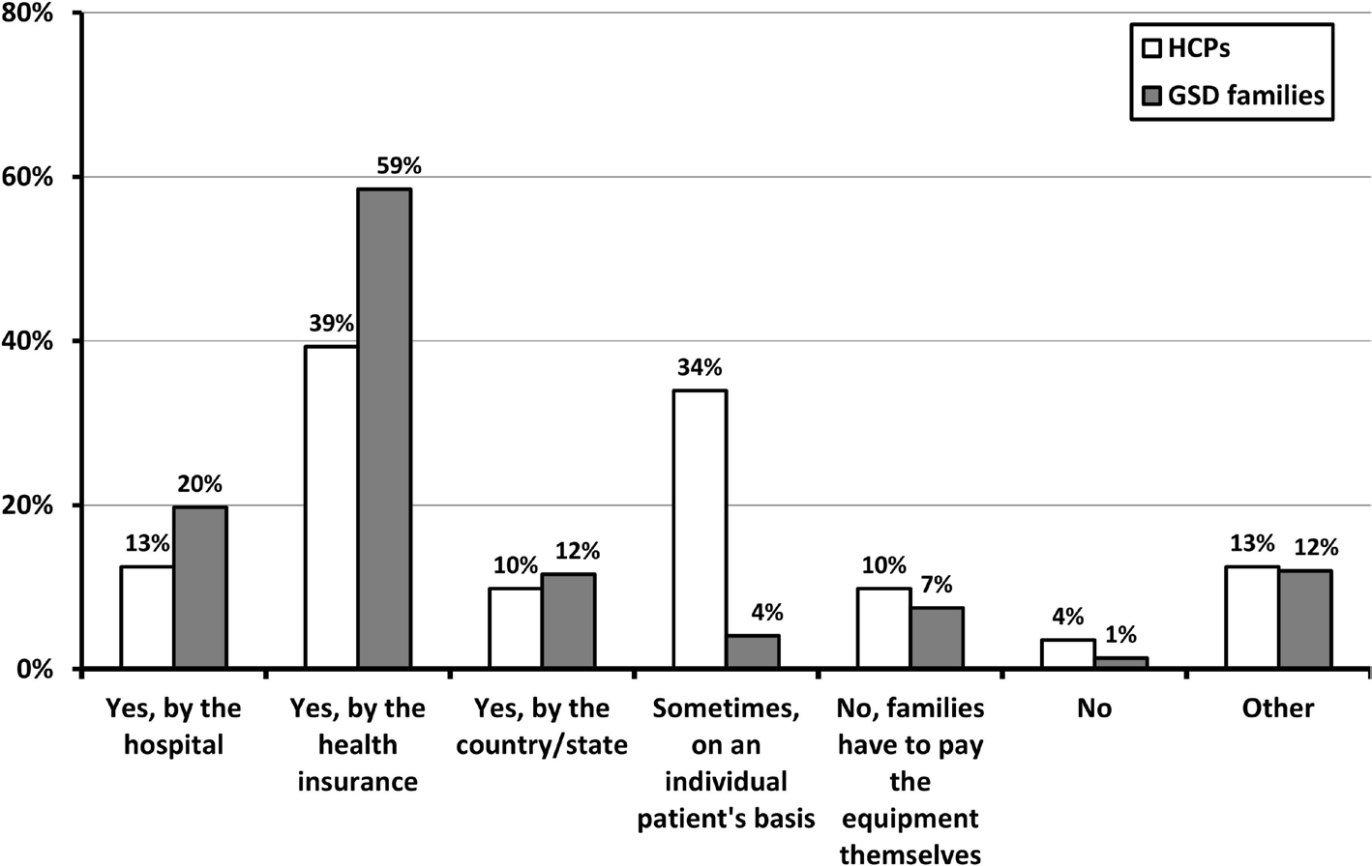
The different ways in which reimbursement of CGM for GSD patients is organized, as reported by HCPs (Q1, question 19) and GSD patients and caregivers (Q2, question 8). The *X*‐axis displays the different ways reimbursement is organized. The *Y*‐axis displays either the percentages as reported by HCPs (in white bars), or the percentages reported by GSD patients and caregivers (grey bars).

The reported mode of reimbursement for CGM was remarkably diverse, as presented by Figure 2. However, 39% (44/112) of the HCPs and more than half of the GSD patients/caregivers (59%; 86/147) answered that reimbursement was provided by health insurances.

**FIGURE 3:**
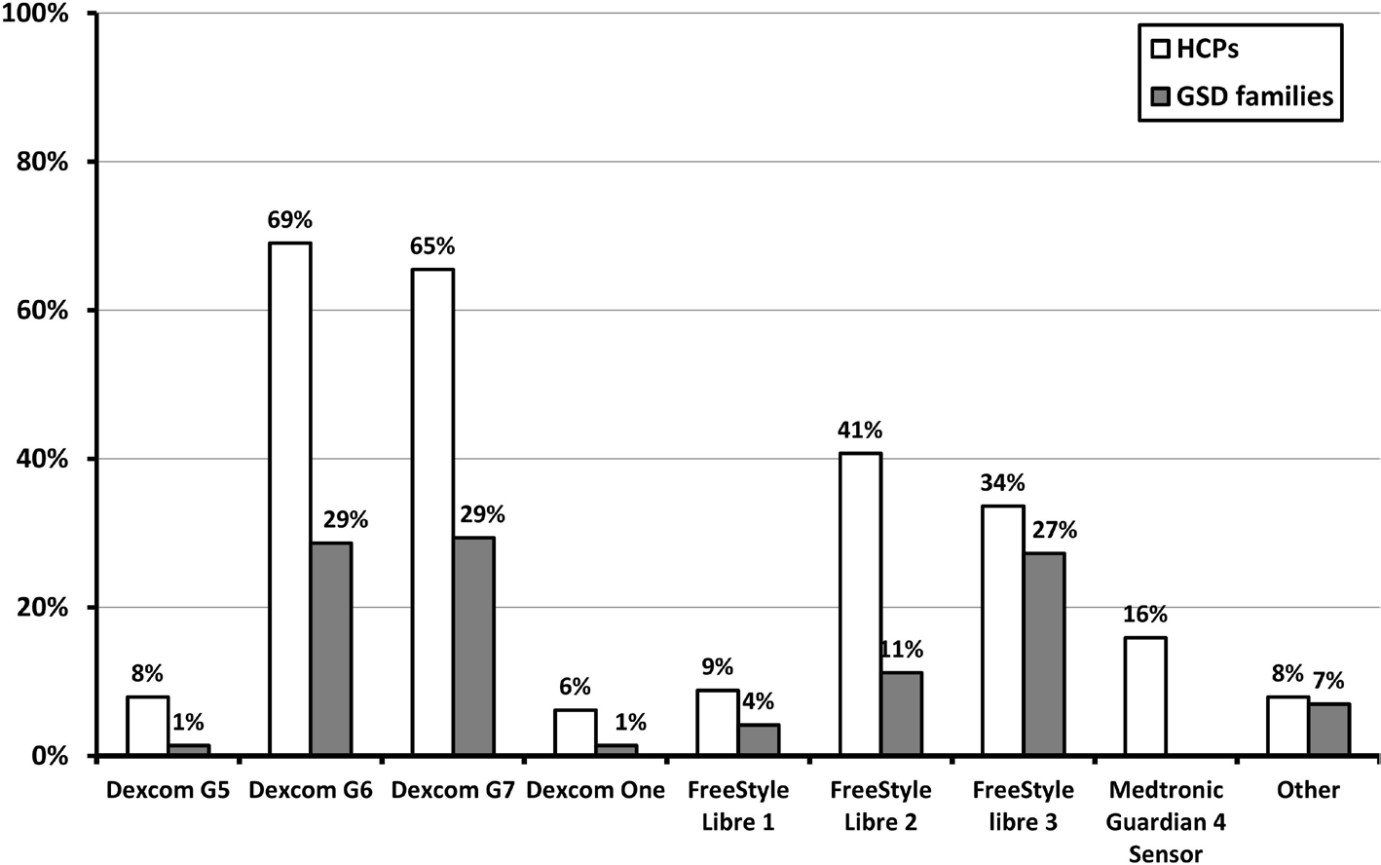
CGM devices used by HCPs (Q1; question 20) and GSD patients and caregivers (Q2; question 9). The *X*‐axis displays different CGM devices. The *Y*‐axis displays either the percentages as reported by HCPs (in white bars), or the percentages reported by GSD patients and caregivers (grey bars).

HCPs and GSD families used at least nine different CGM devices (Figure 3). In most cases, CGM was either used continuously (96/146; 66%) or intermittently (30/146; 21%). In their hospitals, HCPs considered using CGM in patients with liver GSD from the median (range) age of 6 (0–60) months, whereas for families, the median (range) age to consider using CGM was already 2 (0–100) months. Most HCPs (91/114; 80%) recommended using the alarm function of the device.

**FIGURE 4:**
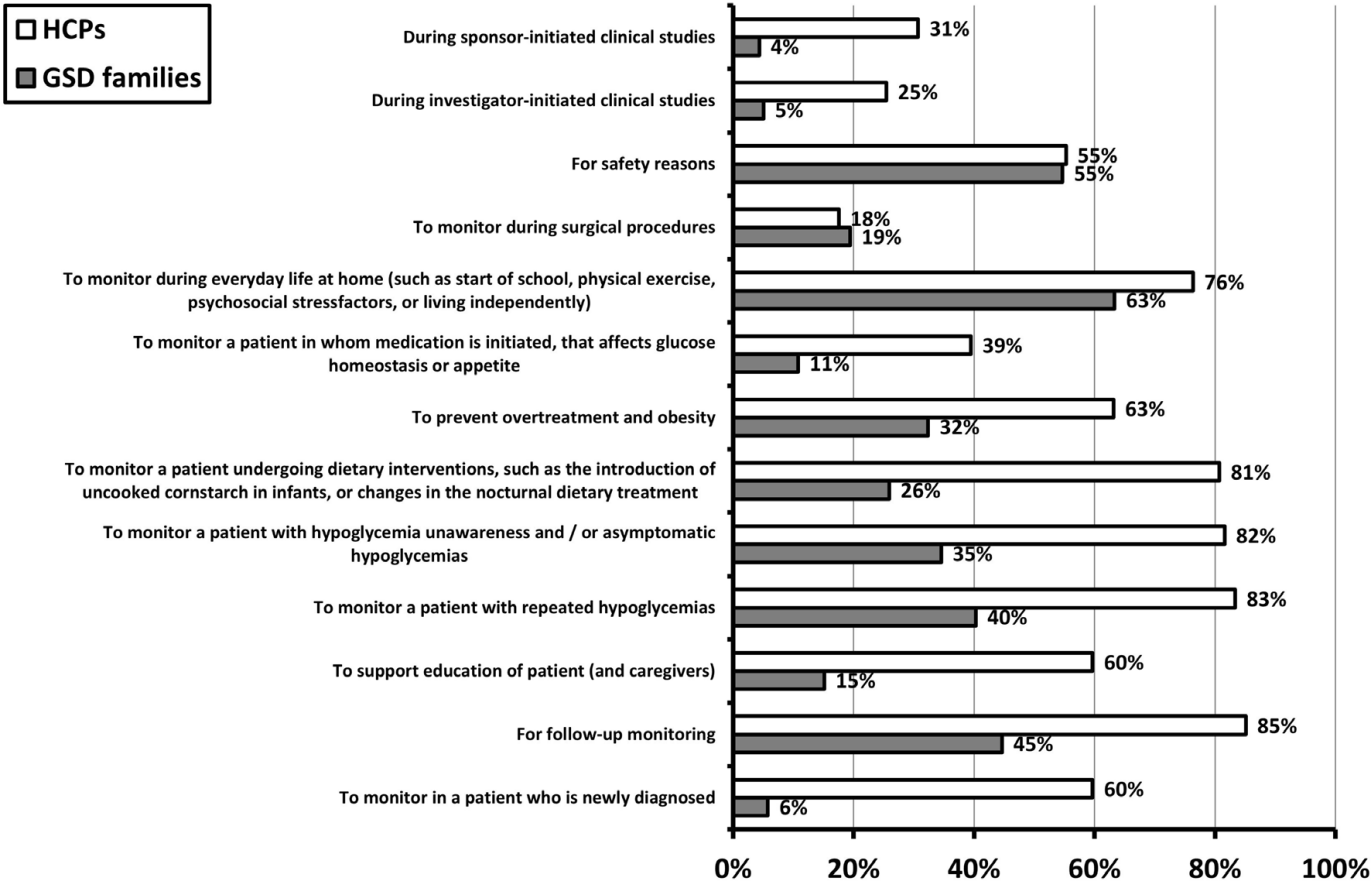
Conditions in which CGM is used as reported by HCPs (Q1; question 21) and GSD patients and caregivers (Q2; question 10). The left panel lists the different situations. The right panel lists the percentages as reported by HCPs (in white bars), or the percentages reported by GSD patients and caregivers (grey bars).

Figure 4 demonstrates CGM use by HCPs and GSD patients/caregivers in different clinical scenarios. Most families use CGM for real‐life daily glucose monitoring and for safety reasons, whereas more than half of the HCPs use it in nine specific clinical settings.

**FIGURE 5:**
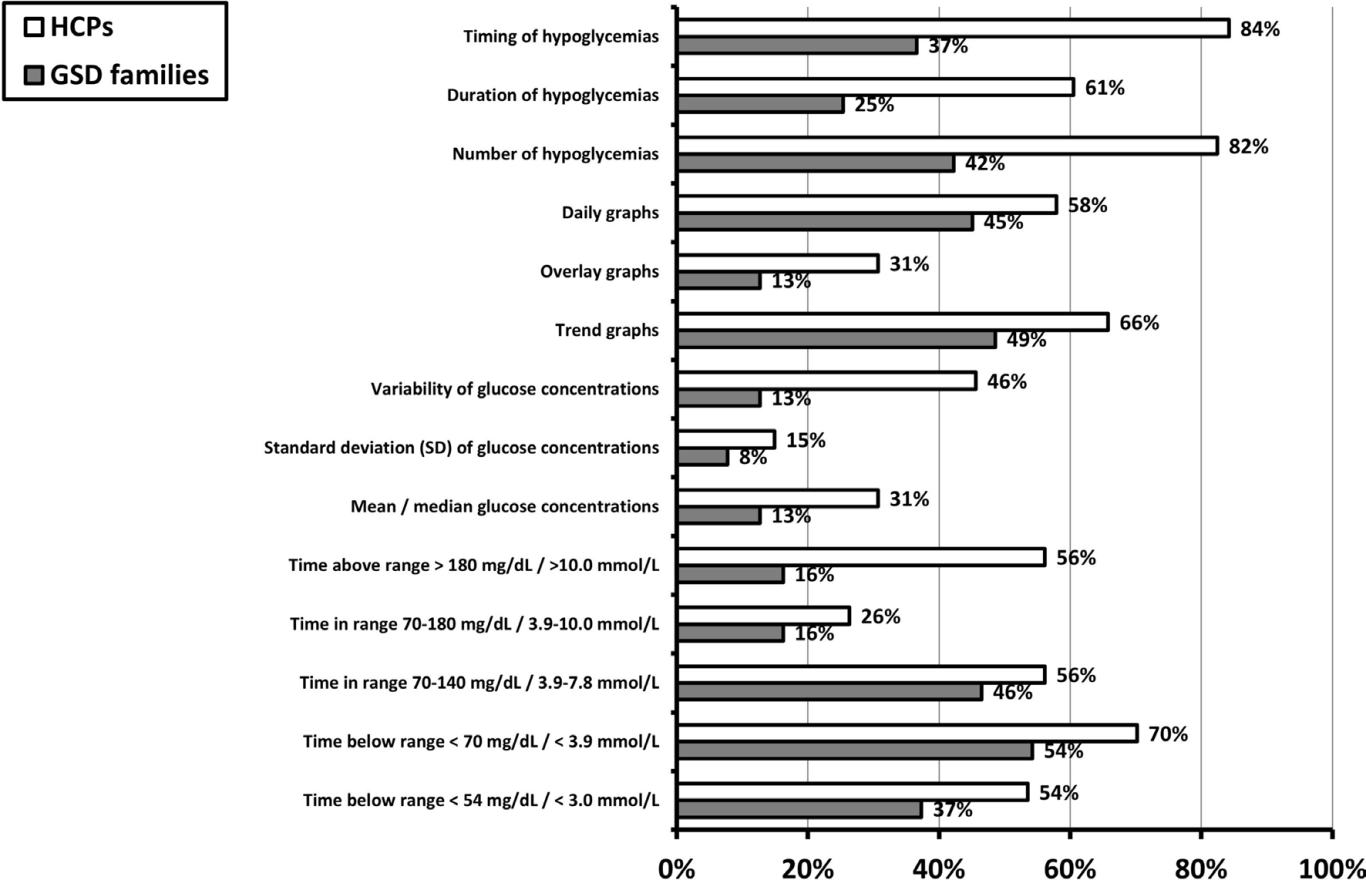
Information used for the assessment of CGM results as reported by HCPs (Q1; question 22) and GSD patients and caregivers (Q2; question 11). The left panel lists the information, including CGM‐metrics. The right panel lists the percentages as reported by HCPs (in white bars), or the percentages reported by GSD patients and caregivers (grey bars).

Figure 5 presents the (combination of) information used by HCPs and GSD patients and caregivers for the assessment of CGM results. Most HCPs made use of the timing (i.e., the hour of the day in which a low glucose event occurs) and number of low glucose events (84% and 82%, respectively), and an additional seven parameters. In contrast, only TBR < 70 mg/dL (< 3.9 mmol/L) was assessed by most of the patients/caregivers.

Table 2 (summary) and File S4 present the categorized advantages and disadvantages of CGM use as reported by HCPs and GSD patients/caregivers.

**TABLE 2:** Summary of advantages and disadvantages of using CGM in liver GSD according to HCPs (Q1, question 29; *n* = 114) and GSD patients and caregivers (Q2, question 15; *n* = 148).

	Advantages	Disadvantages
HCPs	– Monitoring during daily life – Facilitating independence – Fewer finger stick measurements – Increased understanding of blood glucose homeostasis, including responses to meals and (dietary) therapy – Prevention of overtreatment	– Accuracy and reliability issues – Need for ancillary data (e.g., diary, ketone/lactate measurements) – False sense of security – Allergic reactions/skin reactions – Technical Issues – Anxiety – Costs
GSD families	– Improved understanding of disease – Improved awareness of hypoglycemia and hyperglycemia – Continuous aspect of monitoring – Safety (e.g., during illness, sporting activities, overnight feeds) – Ability to have external followers (e.g., caregivers) – Increases independence – Less invasive	– Accuracy and reliability issues – Maintained need for regular monitoring – False sense of security – Placement discomfort including skin irritation/allergic reactions – Developed for diabetes – Lag time of CGM readings – Anxiety/stress (e.g., due to frequent alarms) – Not developed for children < 2 years old – Technical issues – Costs

### 3.3 Consensus Statements Made by Healthcare Providers, Patients and Caregivers on the Utility of CGM in Liver GSD

Based on the respondents' input to Q1 and Q2, we subsequently analyzed the data to assess consensus within and between both groups of respondents (i.e., HCPs, and GSD patients and caregivers, respectively) on statements about the use of CGM in GSD (Table 1). Out of 16 statements used in both questionnaires, HCPs and patient/caregivers reached consensus on 12 statements in both groups. For instance, there was consensus within and between both groups regarding the use of an unblinded CGM where patients/caregivers can immediately see the glucose concentration and share this information with their HCPs. Additionally, CGM was clearly considered standard of care by most HCPs, GSD patients and caregivers. However, in contrast with GSD patients and caregivers, there was no consensus amongst the HCPs regarding the statement “Level 1 hypoglycemia (glucose level of 54–69 mg/dL [3.0–3.9 mmol/L]) assessed by CGM, should be considered an alert threshold independent of any acute symptoms.” GSD patients and their caregivers disagreed with the statement “It is important to assess hyperglycemia after meals or percentage of time‐above‐range.” Interestingly, neither group reached a consensus on the statement “CGM sensor glucose data should also be reported separately for nocturnal (00:00–05:59 h) and daytime periods (06:00–23:59 h).”

### 3.4 Workshop

During the workshop, 30 HCPs participated, 22 of whom had previously answered Q1. The workshop focused on the preliminary results and on the discussion of the three statements for which there was no consensus among HCPs (Table 1; statements with no consensus are in red). It was recognized that the face‐to‐face discussions among experts were valuable to better interpreting these statements. During the workshop it became apparent that it had been unclear for a subset of the participants, whether “alert threshold” also necessitated an intervention. It was discussed that this may have influenced the voting behavior of HCPs in the following statement: “level 1 hypoglycemia (glucose level of 54–69 mg/dL [3.0–3.9 mmol/L]) assessed by CGM, should be considered an alert threshold independent of any acute symptoms.” Additionally, regarding the statement “CGM sensor glucose data should also be reported separately for nocturnal (00:00–05:59 h) and daytime periods (06:00–23:59 h),” several HCPs who did not agree with the statement argued that in reality, the nocturnal timeframe is individualized dependent on age, sleeping behavior and dietary regimen, amongst others. Finally, it was recognized by workshop participants that CGM use may differ depending on GSD subtype and age.

## 4 Discussion

Here, we report the current state‐of‐the‐art use of CGM for the management and monitoring of people with liver GSDs, a group of rare inherited disorders of carbohydrate metabolism. This study among HCPs, GSD patients, and caregivers documents the real‐world experience on CGM use in these rare disorders. The consensus statements are important for stakeholders involved in both routine healthcare (such as physicians, dieticians, nurses, hospital managers, and health insurance companies) and clinical research (such as sponsors of future clinical trials, the European Medicines Agency, the U.S. Food and Drug Administration, and other regulators).

This study indicates that guidelines have been overtaken by daily practice, as CGM systems have seen growing adoption by HCPs and GSD families. After the first FDA‐approval of CGM for DM management in 1999, CGM systems are now routinely used by patients with insulin dependent DM, with recommendations for their use included in major guidelines and consensus statements [14, 15, 16, 17]. In contrast to DM, the application of CGM for patients with rare diseases associated with hypoglycemia (such as liver GSDs) has faced several challenges, which are inherent to the rarity of the conditions. In addition, it has been demonstrated that accuracy and precision of CGM in the hypoglycemia range are generally poorer [5], and devices have gradually improved in that regard [6, 7]. The European guidelines for GSD type Ia [18] and Ib [19] were published in 2002 but did not mention CGM devices. In the more recent GSD I guidelines by the American College of Medical Genetics and Genomics (ACMG; 2014), the application of CGM is only briefly described [20], including the abovementioned concerns regarding lack of accuracy. The ACMG guidelines for GSD III [21], and GSD VI and IX [22] were published in 2010 and 2019, respectively, and do not mention the use of CGM. The recent guidelines for GSD IV recognized that CGM systems are useful in the management of metabolic control in patients with a history of hypoglycemia and/or hyperglycemia [23]. Looking at other rare hypoglycemic conditions, the international guidelines for the diagnosis and management of congenital hyperinsulinism state that “there is not enough current evidence to make a suggestion regarding the use of CGM by subcutaneous sensors” [24].

The present study demonstrates that both HCPs and GSD families report that liver GSD patients benefit from the use of CGM devices and that they should be able to use CGM as a standard of care. Drawbacks of using CGM devices are recognized, including accuracy and reliability issues. Also, in some situations there is a need for additional monitoring, such as ketone measurements. However, both HCPs and GSD families agree that the advantages of CGM in patients with liver GSD outweigh the disadvantages (Table 2 and File S4). In line with previous reports [10, 25], several advantages were explicitly stated. The use of CGM systems in the home environment under real‐life circumstances was mentioned to provide more realistic data and show trends more clearly than SMBG measurements and observations made in the hospital setting. Moreover, the ability to detect asymptomatic hypoglycemia and guidance during diet optimization were indicated as important advantages.

This study reports that CGM in liver GSD serves a different need and purpose than that it was approved for in DM. This is important since CGM allows the detection of asymptomatic hypoglycemia in patients with liver GSDs. Furthermore, HCPs and affected families use CGM at younger ages than recommended. Of note, CGM has been marketed for the management of people with DM from the age of 2 years or 4 years. This difference can be explained by the younger ages at which patients with GSD present and are typically diagnosed. Because of their high glucose requirements and their inability to communicate their symptoms [26], infants and younger children with liver GSDs are at greater risk of acute hypoglycemias. These arguments may explain why CGM in liver GSD is well accepted even at younger ages.

The appreciation of CGM for monitoring liver GSDs is reflected in the high degree of consensus reached among HCPs, patients, and families for most statements (Table 1). Glycemic and metabolic control in GSDs rely heavily on patient self‐management and the use of home medical devices. Therefore, and similar to DM [27], liver GSDs may be ideal rare diseases for developing value‐based telehealth programs [28]. Machine learning, artificial intelligence, and other computer‐generated algorithms have important potential for improving CGM application in hypoglycemic conditions by predicting hypoglycemia to prevent neuroglycopenia and, as a consequence, brain dysfunction [9].

There was no consensus among HCPs on three statements namely, “CGM‐based glycemic targets depend on the liver GSD subtype,” “CGM sensor glucose data should also be reported separately for nocturnal (00:00–05:59 h) and daytime periods (06:00–23:59 h),” and “Level 1 hypoglycemia glucose level of 54–69 mg/dL (3.0–3.9 mmol/L) assessed by CGM, should be considered an alert threshold independent of any acute symptoms.”

There was agreement among HCPs but not among GSD families (87.0% versus 70.8%, respectively) regarding the statement “It is important to assess hyperglycemia after meals or percentage points of time‐above‐range.” It can be hypothesized, that this discrepancy indicates an unmet need for caregiver education regarding the adverse long‐term effects of elevated blood glucose concentrations due to liver GSD‐typical pathophysiologic mechanisms and overtreatment. Hyperglycemia may worsen glycemic control, aggravate hepatic glycogen and lipid storage, and increase the risk of long‐term complications. CGM can be an important tool both for detection and prevention of overtreatment in liver GSDs due to inappropriate amounts of uncooked cornstarch or dietary carbohydrates. This should reduce the risk of developing acute and chronic complications, such as obesity. Future studies are needed to analyze these associations.

There are several reasons why the use of CGM differs between GSDs and DM, although acute hypoglycemia is an important symptom in patients with both conditions. Firstly, DM type 1 and type 2 are common disorders, but each liver GSD subtype meets the definition of a rare disease. Secondly, liver GSDs are managed through (very) detailed medically prescribed diets; there are no registered drugs available for GSD treatment. Thirdly, it is important to acknowledge that the pathophysiology and the hormonal and metabolic counter‐regulatory response during acute hypoglycemia is very different from DM and specific to the GSD subtype. For example, in GSD Ia and GSD Ib, hypoglycemia is associated with hyperlactatemia without elevated ketone body concentrations. In contrast, in the remaining liver GSDs, fasting is typically associated with hyperketosis. Fourthly, carbohydrate requirements in GSD patients change throughout life. For example, decreased cornstarch requirements have been recognized in aging adult GSD I patients [29]. Lastly, hyperglycemia in adults with GSD III [30] or FBS [31] may indicate the development of DM, one of the known long‐term chronic complications in these conditions. Therefore, in our opinion, age, GSD subtype and genotype, diet, physical activity, complications (such as cardiomyopathy and inflammatory bowel disease, in addition to DM that may be present), special circumstances such as pregnancy, social and cultural factors, and cognitive factors (understanding of the patients and caregivers) need to be considered when prescribing the diet and recommending CGM. For GSD management, CGM alarm values, target values and values that require acute action cannot be automatically adopted from existing DM guidelines.

This study has some limitations. Bias may have been introduced due to limited reimbursements and availability of CGM, excluding opinions of patients and HCPs without CGM access. However, the distribution of Q1 ensured that the opinions of important GSD experts could be included. The distribution of Q2 via patient organizations, by which GSD Ib was overrepresented, appears to have resulted in a skewed proportion of respondents compared to the epidemiology of the GSD subtypes. We cannot exclude that individual preferences and differences in device age and usage methods may have influenced current results. Additionally, our approach may have led to sampling bias weighted towards (1) more proactive patients and families, who (2) desire more intervention and closer monitoring, and (3) have a positive perception of CGM. Nonetheless, we believe this is an important study reporting experiences from a large, international group of GSD patient representatives and key opinion leaders.

To conclude, the off‐label use of CGM is now considered standard of care to monitor, recognize, treat, and prevent hypoglycemia in liver GSD. Like narrow therapeutic range drugs, the medically prescribed diets in GSDs have a tight therapeutic window between hypoglycemia and hyperglycemia that requires careful consideration. The use of CGM has allowed patients and providers an avenue aiming to individualize patient care and to better prevent both acute and chronic complications. Country‐specific measures may be required to improve access to CGM for people with liver GSDs. Consensus guidelines are warranted for the use of CGM in liver GSDs, both in routine healthcare settings and in clinical trials. International prospective multicenter studies that correlate CGM metrics with clinically relevant outcomes are crucial, although practically very challenging due to limited possibilities for data sharing and very complex regulations.

## Author Contributions

**Terry G. J. Derks:** conceptualization, data curation, formal analysis, investigation, methodology, project administration, visualization, writing – original draft. **Sarah C. Grünert:** formal analysis, investigation, methodology, writing – original draft. **Ruben J. Overduin:** data curation, formal analysis, investigation, methodology, project administration, visualization, writing – original draft. **Alessandro Rossi:** formal analysis, investigation, methodology, writing – original draft. **CGM GSD Collaborators Group:** investigation, writing – review and editing.

## Conflicts of Interest

Terry G. J. Derks: There are confidentiality agreements with third parties. In the past 36 months, there have been consultation agreements (with Danone S.A., Ultragenyx Pharmaceutical Inc., Moderna Inc., and Beam Therapeutics Inc.), contracts for financial research support for investigator‐initiated research (NCT04311307) and sponsor‐initiated research (NCT03517085, NCT03970278, NCT05139316, and NCT05196165), honoraria for lectures or presentations (by MEDTalks, Prelum, and Danone S.A.), and participations in a Data Safety Monitoring Board (NCT05095727) and Advisory Boards (Ultragenyx Pharmaceutical Inc., Moderna Inc., and Beam Therapeutics Inc.). For all private‐public relationships, all contracts are via UMCG Contract Research Desk and all payments are to UMCG. Sarah C. Grünert: She has received honoraria for educational lectures from Vitaflo GmbH and Ultragenyx Pharmaceutical Inc. as well as for the creation of patient information material for Danone Deutschland GmBH, and received support for attending metabolic expert meetings from Nutricia Metabolics GmbH. She participated in an advisory board for Ultragenyx Pharmaceutical Inc. Ruben J. Overduin: There are confidentiality agreements with third parties. There is a consultation agreement (with Ultragenyx Pharmaceutical Inc.). For all private‐public relationships, all contracts are via UMCG Contract Research Desk and all payments are to UMCG. Alessandro Rossi: There are confidentiality agreements with third parties. In the past 36 months, there have been consultation agreements or honoraria for lectures or presentations with Nestlé, Danone S.A., and Ultragenyx Pharmaceutical Inc.

## Supporting information


**File S1.** SurveyMonkey web‐based questionnaire for HCPs (Q1).
**File S2.** SurveyMonkey web‐based questionnaire for people with liver GSD and caregivers (Q2).
**File S3.** Geographic distribution of questionnaire respondents.
**File S4.** Categorized advantages and disadvantages of using CGM in liver GSD according to HCPs (Q1, question 29; *n* = 114) and GSD patients and caregivers (Q2, question 15; *n* = 148).

## Data Availability

De‐identified survey responses that underlie the results reported in this article are available upon request to the corresponding author.

## Appendix A Members of the CGM GSD Collaborators Group

**Table:**

Name	Surname	Affiliation
Terry G. J.	Derks	Department of Metabolic Diseases, Beatrix Children's Hospital, University Medical Center Groningen, University of Groningen, Groningen, the Netherlands UMCG Center of Expertise for Carbohydrate, Fatty Acid Oxidation and Ketone Bodies Disorders, University Medical Center Groningen, Groningen, the Netherlands
Ruben J.	Overduin	Department of Metabolic Diseases, Beatrix Children's Hospital, University Medical Center Groningen, University of Groningen, Groningen, the Netherlands UMCG Center of Expertise for Carbohydrate, Fatty Acid Oxidation and Ketone Bodies Disorders, University Medical Center Groningen, Groningen, the Netherlands
Bibiana M.	de Oliveira	Medical Genetics Service, Hospital de Clínicas de Porto Alegre, Porto Alegre, Brazil
Julien H.	Park	University Hospital Münster, Department of General Pediatrics, Münster, Germany
David	Weinstein	Department of Pediatrics, University of Connecticut School of Medicine, Farmington, CT, USA
Diva D.	De Leon	Division of Endocrinology and Diabetes, Department of Pediatrics, Children's Hospital of Philadelphia and the University of Pennsylvania Perelman School of Medicine, USA
Yuval E.	Landau	Metabolism Program, Schneider Children's Medical Center of Israel, The Faculty of Medical and Health Sciences, Tel Aviv University, Israel
Alessandro	La Rosa	Paediatric Gastroenterology and Digestive Endoscopy Unit, IRCCS Istituto Giannina Gaslini, Genoa, Italy
Damian	Cohen	Glucolatino, Rosario, Argentina
Anke	Schumann	Department of General Pediatrics, Center for Pediatrics and Adolescent Medicine, University Hospital of Freiburg, Freiburg, Germany
Anne	Kwok Mei Kwun	Hong Kong Children's Hospital
Yunkoo	Kang	Department of Pediatrics, Yonsei University Wonju College of Medicine, Wonju, Republic of Korea
Foekje	de Boer	Department of Metabolic Diseases, Beatrix Children's Hospital, University Medical Center Groningen, University of Groningen, Groningen, the Netherlands UMCG Center of Expertise for Carbohydrate, Fatty Acid Oxidation and Ketone Bodies Disorders, University Medical Center Groningen, Groningen, the Netherlands
María	Clemente	Pediatric Endocrinology Unit, Children's Hospital Vall d'Hebron, Autonomous University of Barcelona and Center for Biomedical Research on Rare Diseases, Barcelona, Spain
Wendy E.	Smith	The Barbara Bush Children's Hospital at Maine Medical Center, Portland, ME, USA
Margo Sheck	Breilyn	Department of Genetics and Genomic Sciences, Icahn School of Medicine at Mount Sinai, New York, NY, USA
Rebecca	Riba‐Wolman	Department of Pediatrics, University of Connecticut Medical School, Farmington, CT, USA; Pediatric Endocrinology, Connecticut Children's Medical Center, Hartford, CT, USA
Karen	Loechner	Department of Pediatrics, University of Connecticut Medical School, Farmington, CT, USA; Pediatric Endocrinology, Connecticut Children's Medical Center, Hartford, CT, USA
Malaya	Mount	Department of Pediatrics, University of Connecticut Medical School, Farmington, CT, USA; Pediatric Endocrinology, Connecticut Children's Medical Center, Hartford, CT, USA
John	Mitchell	Division of pediatric endocrinology, McGill University Health Center, Montreal, Canada
Samantha	Vergano	Division of Genetic Medicine, Seattle Children's Hospital, Seattle WA USA; Department of Pediatrics, University of Washington School of Medicine, Seattle WA USA
Stefanie	Rosenbaum‐Fabian	Department of General Pediatrics, Adolescent Medicine and Neonatology, Medical Center–University of Freiburg, Faculty of Medicine, Freiburg, Germany
Shaylee	King	Department of Pediatrics, University of Connecticut Medical School, Farmington, CT, USA; Pediatric Endocrinology, Connecticut Children's Medical Center, Hartford, CT, USA
Iris	Ferrecchia	Glycogen Storage Disease Program, Connecticut Children's Medical Center, Hartford, Connecticut, USA (retired)
Kaustuv	Bhattacharya	Genetic Metabolic Disorders Service, Sydney Children's Hopitals' Network, Sydney, New South Wales, Australia
David F.	Rodriguez‐Buritica	Department of Pediatrics, Division of Medical Genetics, McGovern Medical School at the University of Texas Health; Science Center at Houston (UTHealth Houston) and Children's Memorial Hermann Hospital, Houston, Texas, USA
Katalin	Ross	Glycogen Storage Disease Program, Connecticut Children's, Hartford, CT, USA
Anastasia	Skouma	Institouto Ygeias Tou Paidiou, Institute of Child Health, Athens, Greece
Allan M.	Lund	Center for Inherited Metabolic Diseases, Departments of Pediatrics and Clinical Genetics, Copenhagen University Hospital, Rigshospitalet, Copenhagen, Denmark
Elizabeth	Robinson	Department of Pediatric Gastroenterology, Hepatology, and Nutrition Cleveland Clinic Foundation Cleveland Ohio USA
Caitie	Hessenthaler	Children's Wisconsin, Milwaukee, WI, USA
Emma	Corcoran	National Centre for Inherited Metabolic Disorders, Mater Misericordiae University Hospital, D07 R2WY Dublin, Ireland
Tassia	Tonon	Programa de Pós‐graduação em Ciências Médicas, Universidade Federal do Rio Grande do Sul–UFRGS–Porto Alegre (RS), Brasil
Daniela	Karall	Clinic for Pediatrics, Division of Inherited Metabolic Disorders, Medical University of Innsbruck, Innsbruck, Austria
Laura	Sliwoski	Department of Molecular and Medical Genetics, Oregon Health & Science University, Portland, Oregon, USA
Ulrike	Steuerwald	Medical Center, National Hospital System, Torshavn, Faroe Islands
Patricia	Pérez Mohand	Department of Pediatrics, Hospital Universitario 12 de Octubre, Madrid, Spain
Julieta	Bonvin Sallago	Glycogen Storage Disease Program, Connecticut Children's Medical Center, Hartford, Connecticut, USA; Department of Pediatrics, University of Connecticut Medical School, Farmington, CT, USA
Nerea	López Maldonado	Asociación Española de Enfermos de Glucogenosis (AEEG), Spain CAP Rambla, Fundación Asistencial MutuaTerrrassa, Terrassa, Barcelona, Spain
Renee	Bethel	Arkansas Children's, AR, USA
Monica	Boyer	Division of Metabolic Disorders, CHOC Children's Hospital, Orange, CA, United States of America
Timothy	Fazio	Metabolic Diseases Unit, Royal Melbourne Hospital, Parkville, Victoria, Australia; Department of Medicine, Melbourne Medical School, the University of Melbourne, Parkville, Victoria, Australia
Vincenza	Gragnaniello	Division of Inherited Metabolic Diseases, Department of Women and Children's Health, University Hospital, Padua, Italy
María‐Luz	Couce	Hospital Clínico Universitario de Santiago, University of Santiago de Compostela, IDIS, CIBERER, A Coruña, Spain
Chiara	Falanga	Department of General Pediatrics, Adolescent Medicine and Neonatology, Medical Centre‐ University of Freiburg, Faculty of Medicine, Freiburg, Germany
Urh	Groselj	UMC–University Children's Hospital Ljubljana, University of Ljubljana, Faculty of Medicine
Brandy	Rawls‐Castillo	Department of Molecular and Human Genetics Baylor College of Medicine Houston Texas USA
Zazil	Olivares‐Sandoval	Unidad de Genética de la Nutrición. Instituto Nacional de Pediatría, Secretaría de Salud‐Instituto de Investigaciones Biomédicas, Universidad Nacional Autónoma de México, Mexico City, Mexico
Carolina	F. Moura de Souza	Medical Genetics Service, Hospital de Clínicas de Porto Alegre, Porto Alegre, RS, Brazil
Sabine	Scholl‐Buergi	Department of Pediatrics I, Medical University of Innsbruck, 6020 Innsbruck, Austria
Annalisa	Madeo	Paediatric Gastroenterology, Hepatology and Digestive Endoscopy Unit, IRCCS Istituto Giannina Gaslini, Genoa, Italy
Julia B.	Hennermann	Center for Pediatric and Adolescent Medicine Villa Metabolica, University Medical Center Mainz, Germany
Eva	Venegas	Endocrinology and Nutrition Department, Hospital Universitario Virgen del Rocío, Sevilla, Spain
Arianna	Maiorana	Division of Metabolic Diseases and Hepatology, Ospedale Pediatrico Bambino Gesù, IRCCS, Rome, Italy
Dorothea	Haas	Heidelberg University, Medical Faculty of Heidelberg, Center for Child and Adolescent Medicine, Division of Child Neurology and Metabolic Medicine, Heidelberg, Germany
Benjamin	Weil	Northwell Health Medical Genetics, Great Neck, NY, USA
Eva	Thimm	Department of General Paediatrics, Neonatology and Paediatric Cardiology, University Children's Hospital, Heinrich Heine University Düsseldorf, Düsseldorf, Germany
Alboren	Shtylla	Department of Endocrinology and Metabolism, Heidelberg University Hospital, Heidelberg, Germany
Thomas	Opladen	Division of Pediatric Neurology and Metabolic Medicine, Department I, Center for Pediatric and Adolescent Medicine, Medical Faculty Heidelberg, Heidelberg University
Thomas	Scherer	Department of Medicine III, Division of Endocrinology and Metabolism, Medical University of Vienna
Danielle K.	Bourque	Division of Metabolics and Newborn Screening, Department of Pediatrics, CHEO, Ottawa, Canada
Tergita	Preci	Rare Metabolic Disorders Centre Heidelberg, Germany
Michel	Hochuli	Department of Diabetes, Endocrinology, Nutritional Medicine and Metabolism, Bern University Hospital and University of Bern, Switzerland
Thomas	Casswall	Pediatric Gastroenterology, Hepatology, and Nutrition Karolinska University Hospital, and CLINTEC, Karolinska Institutet, Stockholm, Sweden
Ellen	Crushell	National Centre for Inherited Metabolic Disorders, Children's Health Ireland at Temple Street, Dublin, Ireland
Shagun	Kaur	Division of Genetics and Metabolism, Phoenix Children's, Arizona, USA
Petra	Zsidegh	Semmelweis University Pediatric Centre, Newborn Screening and Metabolic Centre, Budapest, Hungary
Risto	Lapatto	New Children's Hospital, Pediatric Research Center, University of Helsinki and Helsinki University Hospital, Helsinki, Finland
Matthias	Gautschi	Division of Paediatric Endocrinology, Diabetology and Metabolism, Department of Paediatrics, and Institute of Clinical Chemistry, Inselspital, Bern University Hospital, University of Bern, Switzerland
Dorothea	Möslinger	Department of Paediatrics and Adolescent Medicine, Medical University of Vienna, Vienna, Austria
Peter	Witters	Center for Metabolic Diseases, Department of Paediatrics, University Hospitals Leuven, Leuven, Belgium Department of Development and Regeneration, KU Leuven, Leuven, Belgium
Ute	Stachelhaus‐Theimer	Glykogenose Deutschland e.V., Am Römerberg 33e, 55 270 Essenheim. Germany
Flannery	Tomberlin	Metabolic RD–A member of GMDI and the listserv
Ana	Drole Torkar	Department of Endocrinology, Diabetes, and Metabolic Diseases, University Children's Hospital, University Medical Centre Ljubljana, Ljubljana, Slovenia
Elena	Procopio	Metabolic and Muscular Unit–Meyer Children's Hospital IRCCS‐Florence‐Italy
Luisa	Diogo Matos	Reference Center of Inherited Metabolic Diseases Hospital Pediátrico –Unidade Local de Saúde de Coimbra, Portugal
Saskia	Wortmann	Salzburger Landeskliniken (SALK) and Paracelsus Medical University (PMU), Salzburg, Austria
Julia	Neugebauer	Department of Paediatric Gastroenterology, Nephrology and Metabolic Medicine. Charité – Universitaetsmedizin, Berlin (13353), Berlin, Germany. Center for Chronically Sick Children, Charité—Universitaetsmedizin Berlin (13353), Berlin, Germany
Teresa	Rink	Genomic Medicine Program, Children's Minnesota, Minneapolis, MN, USA
Natalie	Weinhold	Department of Pediatric Gastroenterology, Nephrology and Metabolic Medicine, Center for Chronically Sick Children, Charité‐Universitätsmedizin Berlin, Germany
Suresh	Vijay	Pediatrics, Birmingham Children's Hospital NHS Foundation Trust, UK
Krista	Engen	University of Illinois at Chicago, IL, USA
Jean‐Marc	Nuoffer	University Institute of Clinical Chemistry, University of Bern, Bern, Switzerland
Andrea	Haijer‐Schreuder	Department of Metabolic Diseases, Beatrix Children's Hospital, University Medical Center Groningen, University of Groningen, Groningen, the Netherlands UMCG Center of Expertise for Carbohydrate, Fatty Acid Oxidation and Ketone Bodies Disorders, University Medical Center Groningen, Groningen, the Netherlands
Peter	Freisinger	Children's Hospital Reutlingen, Klinikum am Steinenberg Reutlingen, Reutlingen, Germany
Monika	Williams	Duke University, Durham, NC, USA
Patricia	Janeiro	Lisbon Reference Centre for metabolic diseases, ULSSM, Portugal
Justė	Parnarauskienė	Pediatric Center, Vilnius university hospital Santaros klinikos, Vilnius, Lithuania
Çiğdem Seher	Kasapkara	Ankara Yıldırım Beyazıt University, Ankara Bilkent City Hospital, Department of Pediatric Metabolism, Ankara/Turkey
Miguel Angel	Martinez Olmos	Division of Endocrinology and Nutrition, University Hospital of Santiago de Compostela, University of Santiago de Compostela, IDIS, CIBEROBN, A Coruña, Spain
Areeg	El‐Gharbawy	Division of Medical Genetics and Genomics, Department of Pediatrics, Duke University Medical Center, Durham, NC, USA
Heather	Saavedra	Department of Pediatrics, Division of Medical Genetics, McGovern Medical School at the University of Texas Health Science Center at Houston (UTHealth Houston) and Children's Memorial Hermann Hospital, Houston, Texas, USA
Nicola	Longo	Division of Clinical Genetics, Department of Human Genetics, University of California Los Angeles, Los Angeles, CA, USA
Magali	Reyes Apodaca	Unidad de Investigación y Diagnóstico en Nefrología y Metabolismo Mineral Óseo, Hospital Infantil de México Federico Gómez, CDMX, México
Sunita	Bijarnia‐Mahay	Institute of Medical Genetics & Genomics, Sir Ganga Ram Hospital, New Delhi, India
Leyla	Tümer	Department of Paediatric Metabolism and Nutrition, Gazi University School of Medicine, Emniyet Street, Yenimahalle, Ankara, Turkey
Martina	Huemer	Division of Metabolism, Children's Research Centre, University Children's, Hospital Zurich, University of Zurich, Steinwiesstrasse 75, Zürich, 8032, Switzerland; Department of Paediatrics, LKH Bregenz, Bregenz 6900, Austria
René	Santer	Department of Pediatrics, University Medical Center Eppendorf, Hamburg, Germany
Lina	Ghaloul‐Gonzalez	Division of Genetic and Genomic Medicine, Department of Pediatrics, University of Pittsburgh School of Medicine, Pittsburgh, Pennsylvania, USA Department of Human Genetics, Graduate School of Public Health, University of Pittsburgh, Pittsburgh, Pennsylvania, USA
Katherine	Anderson	Department of Pediatrics, Division of Clinical Genetics, University of Vermont, Burlington, VT, USA
Rebecca L.	Koch	Department of Pediatrics, Division of Medical Genetics, Duke University Medical Center, 905 LaSalle St., Durham, NC, USA
Christian	Kogelmann	University Medical Centre Hamburg‐Eppendorf, Germany
Annamaria	Sapuppo	Unit of Pediatrics and Pediatric Emergency Department, University Hospital Policlinico “G.Rodolico‐S.Marco”, 95 123 Catania, Italy
Brian	Shayota	Division of Medical Genetics, Department of Pediatrics, University of Utah, Salt Lake City, UT, USA
Melanie M.	van der Klauw	Department of Endocrinology, University of Groningen, University Medical Center Groningen, Groningen, the Netherlands UMCG Center of Expertise for Carbohydrate, Fatty Acid Oxidation and Ketone Bodies Disorders, University Medical Center Groningen, Groningen, the Netherlands
Fatih	Ezgu	Gazi University Faculty of Medicine, Departments of Pediatric Metabolism and Genetics, Ankara, Turkey
Clemens	Kamrath	Department of General Pediatrics, Adolescent Medicine and Neonatology, Medical Centre–University of Freiburg, Faculty of Medicine, Freiburg, Germany
Camilla	Carøe	Center for Inherited Metabolic Diseases, Departments of Pediatrics, Copenhagen University Hospital, Rigshospitalet, Copenhagen, Denmark
Karolina M.	Stepien	Adult Inherited Metabolic Diseases Department, Salford Royal Organization, Northern Care Alliance NHS Foundation Trust, Salford, UK
Fiona	White	Manchester University NHS Foundation Trust, Manchester, UK
Sarah C.	Grünert	Department of General Pediatrics, Adolescent Medicine and Neonatology, Medical Centre–University of Freiburg, Faculty of Medicine, Freiburg, Germany
Alessandro	Rossi	Department of Translational Medicine, Section of Pediatrics, University of Naples “Federico II”, Naples, Italy

## References

[1] W. B. Hannah , T. G. J. Derks , M. L. Drumm , S. C. Grünert , P. S. Kishnani , and J. Vissing , “Glycogen Storage Diseases,” Nature Reviews. Disease Primers 9, no. 1 (2023): 46, 10.1038/S41572-023-00456-Z. 37679331

[2] T. Danne , R. Nimri , T. Battelino , et al., “International Consensus on Use of Continuous Glucose Monitoring,” Diabetes Care 40, no. 12 (2017): 1631–1640, 10.2337/dc17-1600. 29162583 PMC6467165

[3] T. Battelino , T. Danne , R. M. Bergenstal , et al., “Clinical Targets for Continuous Glucose Monitoring Data Interpretation: Recommendations From the International Consensus on Time in Range,” Diabetes Care 42, no. 8 (2019): 1593–1603, 10.2337/dci19-0028. 31177185 PMC6973648

[4] T. Battelino , C. M. Alexander , S. A. Amiel , et al., “Continuous Glucose Monitoring and Metrics for Clinical Trials: An International Consensus Statement,” Lancet Diabetes and Endocrinology 11, no. 1 (2023): 42–57, 10.1016/S2213-8587(22)00319-9. 36493795

[5] D. Rodbard , “Characterizing Accuracy and Precision of Glucose Sensors and Meters,” Journal of Diabetes Science and Technology 8, no. 5 (2014): 980–985, 10.1177/1932296814541810. 25037194 PMC4455380

[6] L. Heinemann , M. Schoemaker , G. Schmelzeisen‐Redecker , et al., “Benefits and Limitations of MARD as a Performance Parameter for Continuous Glucose Monitoring in the Interstitial Space,” Journal of Diabetes Science and Technology 14, no. 1 (2020): 135–150, 10.1177/1932296819855670. 31216870 PMC7189145

[7] S. Alva , T. Bailey , R. Brazg , et al., “Accuracy of a 14‐Day Factory‐Calibrated Continuous Glucose Monitoring System With Advanced Algorithm in Pediatric and Adult Population With Diabetes,” Journal of Diabetes Science and Technology 16, no. 1 (2022): 70–77, 10.1177/1932296820958754. 32954812 PMC8875061

[8] N. Patience , A. Sheehan , C. Cummings , and M. E. Patti , “Medical Nutrition Therapy and Other Approaches to Management of Post‐Bariatric Hypoglycemia: A Team‐Based Approach,” Current Obesity Reports 11, no. 4 (2022): 277–286, 10.1007/s13679-022-00482-0. 36074258

[9] C. Worth , M. Dunne , A. Ghosh , S. Harper , and I. Banerjee , “Continuous Glucose Monitoring for Hypoglycaemia in Children: Perspectives in 2020,” Pediatric Diabetes 21, no. 5 (2020): 697–706, 10.1111/pedi.13029. 32315515

[10] F. Peeks , I. J. Hoogeveen , R. L. Feldbrugge , et al., “A Retrospective In‐Depth Analysis of Continuous Glucose Monitoring Datasets for Patients With Hepatic Glycogen Storage Disease: Recommended Outcome Parameters for Glucose Management,” Journal of Inherited Metabolic Disease 44, no. 5 (2021): 1136–1150, 10.1002/JIMD.12383. 33834518 PMC8519135

[11] A. Rossi , A. Venema , P. Haarsma , et al., “A Prospective Study on Continuous Glucose Monitoring in Glycogen Storage Disease Type Ia: Toward Glycemic Targets,” Journal of Clinical Endocrinology and Metabolism 107, no. 9 (2022): 3612–3623, 10.1210/clinem/dgac411. PMC9387687 35786777

[12] C. Worth , L. Hoskyns , M. Salomon‐Estebanez , et al., “Continuous Glucose Monitoring for Children With Hypoglycaemia: Evidence in 2023,” Frontiers in Endocrinology 14 (2023): 1116864, 10.3389/fendo.2023.1116864. 36755920 PMC9900115

[13] F. Peeks , W. F. Boonstra , L. de Baere , et al., “Research Priorities for Liver Glycogen Storage Disease: An International Priority Setting Partnership With the James Lind Alliance,” Journal of Inherited Metabolic Disease 43, no. 2 (2020): 279–289, 10.1002/JIMD.12178. 31587328 PMC7079148

[14] NICE guideline , “Type 1 Diabetes in Adults: Diagnosis and Management,” (2022), https://www.nice.org.uk/guidance/ng17.

[15] N. A. Elsayed , G. Aleppo , V. R. Aroda , et al., “7. Diabetes Technology: Standards of Care in Diabetes—2023,” Diabetes Care 46, no. suppl 1 (2023): S111–S127, 10.2337/dc23-S007. 36507635 PMC9810474

[16] R. I. G. Holt , J. H. Devries , A. Hess‐Fischl , et al., “The Management of Type 1 Diabetes in Adults. A Consensus Report by the American Diabetes Association (ADA) and the European Association for the Study of Diabetes (EASD),” Diabetes Care 44, no. 11 (2021): 2589–2625, 10.2337/dci21-0043. 34593612

[17] A. Neu , J. Bürger‐Büsing , T. Danne , et al., “Diagnosis, Therapy and Follow‐Up of Diabetes Mellitus in Children and Adolescents,” Experimental and Clinical Endocrinology & Diabetes 127, no. S 01 (2019): S39–S72, 10.1055/a-1018-8963. 31860926

[18] J. P. Rake , G. Visser , P. Labrune , J. V. Leonard , K. Ullrich , and G. P. A. Smit , “Guidelines for Management of Glycogen Storage Disease Type I ‐ European Study on Glycogen Storage Disease Type I (ESGSD I),” European Journal of Pediatrics 161 (2002): S112–S119, 10.1007/s00431-002-1016-7. 12373584

[19] G. Visser , J. Rake , P. Labrune , et al., “Consensus Guidelines for Management of Glycogen Storage Disease Type 1b ‐ European Study on Glycogen Storage Disease Type 1,” European Journal of Pediatrics 161 Suppl 1 (2002): S120–S123, 10.1007/s00431-002-1017-6. 12373585

[20] P. S. Kishnani , S. L. Austin , J. E. Abdenur , et al., “Diagnosis and Management of Glycogen Storage Disease Type I: A Practice Guideline of the American College of Medical Genetics and Genomics,” Genetics in Medicine 16, no. 11 (2014): 1–29, 10.1038/GIM.2014.128. 25356975

[21] P. S. Kishnani , S. L. Austin , P. Arn , et al., “Glycogen Storage Disease Type III Diagnosis and Management Guidelines,” Genetics in Medicine 12, no. 7 (2010): 446–463, 10.1097/GIM.0b013e3181e655b6. 20631546

[22] P. S. Kishnani , J. Goldstein , S. L. Austin , et al., “Diagnosis and Management of Glycogen Storage Diseases Type VI and IX: A Clinical Practice Resource of the American College of Medical Genetics and Genomics (ACMG),” Genetics in Medicine 21, no. 4 (2019): 772–789, 10.1038/s41436-018-0364-2. 30659246

[23] R. L. Koch , C. Soler‐Alfonso , B. T. Kiely , et al., “Diagnosis and Management of Glycogen Storage Disease Type IV, Including Adult Polyglucosan Body Disease: A Clinical Practice Resource,” Molecular Genetics and Metabolism 138, no. 3 (2023): 107525, 10.1016/j.ymgme.2023.107525. 36796138

[24] D. D. De Leon , J. B. Arnoux , I. Banerjee , et al., “International Guidelines for the Diagnosis and Management of Hyperinsulinism,” Hormone Research in Pædiatrics 97, no. 3 (2024): 279–298, 10.1159/000531766. PMC11124746 37454648

[25] D. S. Bali , A. El‐Gharbawy , S. Austin , S. Pendyal , and P. S. Kishnani , “Glycogen Storage Disease Type I,” in GeneReviews (University of Washington, 2021), https://www.ncbi.nlm.nih.gov/books/NBK1312/. 20301489

[26] D. M. Bier , R. D. Leake , M. W. Haymond , et al., “Measurement of “True” Glucose Production Rates in Infancy and Childhood With 6,6‐Dideuteroglucose,” Diabetes 26, no. 11 (1977): 1016–1023, 10.2337/diab.26.11.1016. 913891

[27] S. Crossen , J. Raymond , and A. Neinstein , “Top 10 Tips for Successfully Implementing a Diabetes Telehealth Program,” Diabetes Technology & Therapeutics 22, no. 12 (2020): 920–928, 10.1089/dia.2020.0042. 32191141 PMC7757601

[28] I. J. Hoogeveen , F. Peeks , F. de Boer , et al., “A Preliminary Study of Telemedicine for Patients With Hepatic Glycogen Storage Disease and Their Healthcare Providers: From Bedside to Home Site Monitoring,” Journal of Inherited Metabolic Disease 41, no. 6 (2018): 929–936, 10.1007/s10545-018-0167-2. 29600495 PMC6326981

[29] K. R. Dahlberg , I. A. Ferrecchia , M. Dambska‐Williams , et al., “Cornstarch Requirements of the Adult Glycogen Storage Disease Ia Population: A Retrospective Review,” Journal of Inherited Metabolic Disease 43, no. 2 (2020): 269–278, 10.1002/jimd.12160. 31415093

[30] C. P. Sentner , I. J. Hoogeveen , D. A. Weinstein , et al., “Glycogen Storage Disease Type III: Diagnosis, Genotype, Management, Clinical Course and Outcome,” Journal of Inherited Metabolic Disease 39, no. 5 (2016): 697–704, 10.1007/s10545-016-9932-2. 27106217 PMC4987401

[31] S. Sharari , M. Abou‐Alloul , K. Hussain , and F. A. Khan , “Fanconi–Bickel Syndrome: A Review of the Mechanisms That Lead to Dysglycaemia,” International Journal of Molecular Sciences 21, no. 17 (2020): 6286, 10.3390/ijms21176286. 32877990 PMC7504390

