# Supplementary material for: State of the Art and Consensus Statements by Healthcare Providers, Patients, and Caregivers on Continuous Glucose Monitoring in Liver Glycogen Storage Diseases
Source: J Inherit Metab Dis. 2025 May 13;48(3):e70040. doi: 10.1002/jimd.70040 (PMC12074895; doi:10.1002/jimd.70040)
Supplement: Supplementary file 1 — File S1. SurveyMonkey web‐based questionnaire for HCPs (Q1). File S2. SurveyMonkey web‐based questionnaire for people with liver GSD and caregivers (Q2). File S3. Geographic distribution of questionnaire respondents. File S4. Categorized advantages and disadvantages of using CGM in liver GSD according to HCPs (Q1, question 29; n = 114) and GSD patients and caregivers (Q2, question 15; n = 148). [file JIMD-48-0-s001.docx]

**Supplementary files**

**Table of contents**

[Supplementary File 1. SurveyMonkey® web-based questionnaire for HCPs (Q1). 2](#_Toc194437333)

[Supplementary File 2. SurveyMonkey® web-based questionnaire for people with liver GSD and caregivers (Q2). 18](#_Toc194437334)

[Supplementary File 3. Geographic distribution of questionnaire respondents. 30](#_Toc194437335)

[Supplementary File 4. Categorized advantages and disadvantages of using CGM in liver GSD according to HCPs (Q1, question 29; n=114) and GSD patients and caregivers (Q2, question 15; n=148). 31](#_Toc194437336)

# **Supplementary File 1. SurveyMonkey® web-based questionnaire for HCPs (Q1).**

Please see the following pages for the printout version of the online survey.


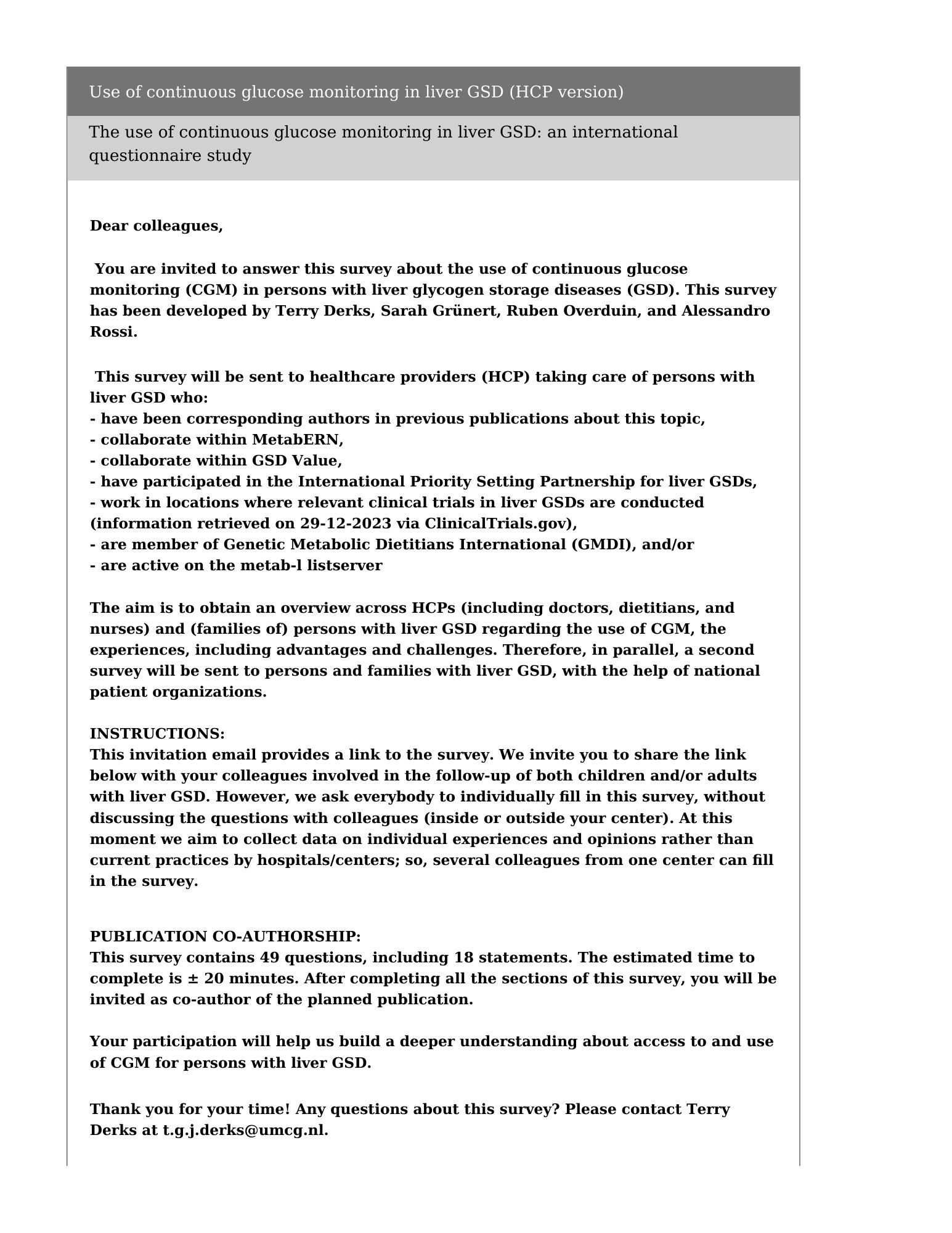

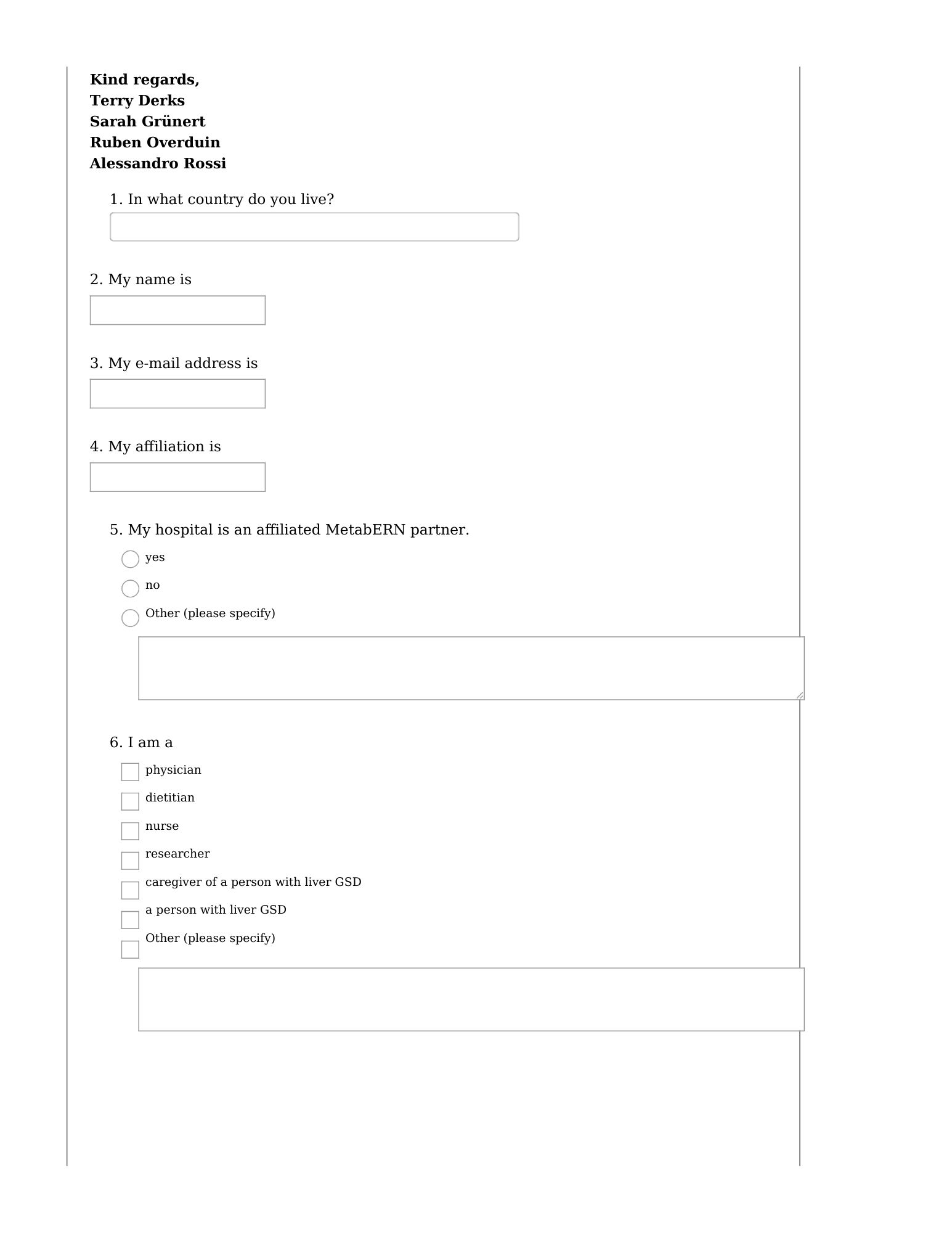

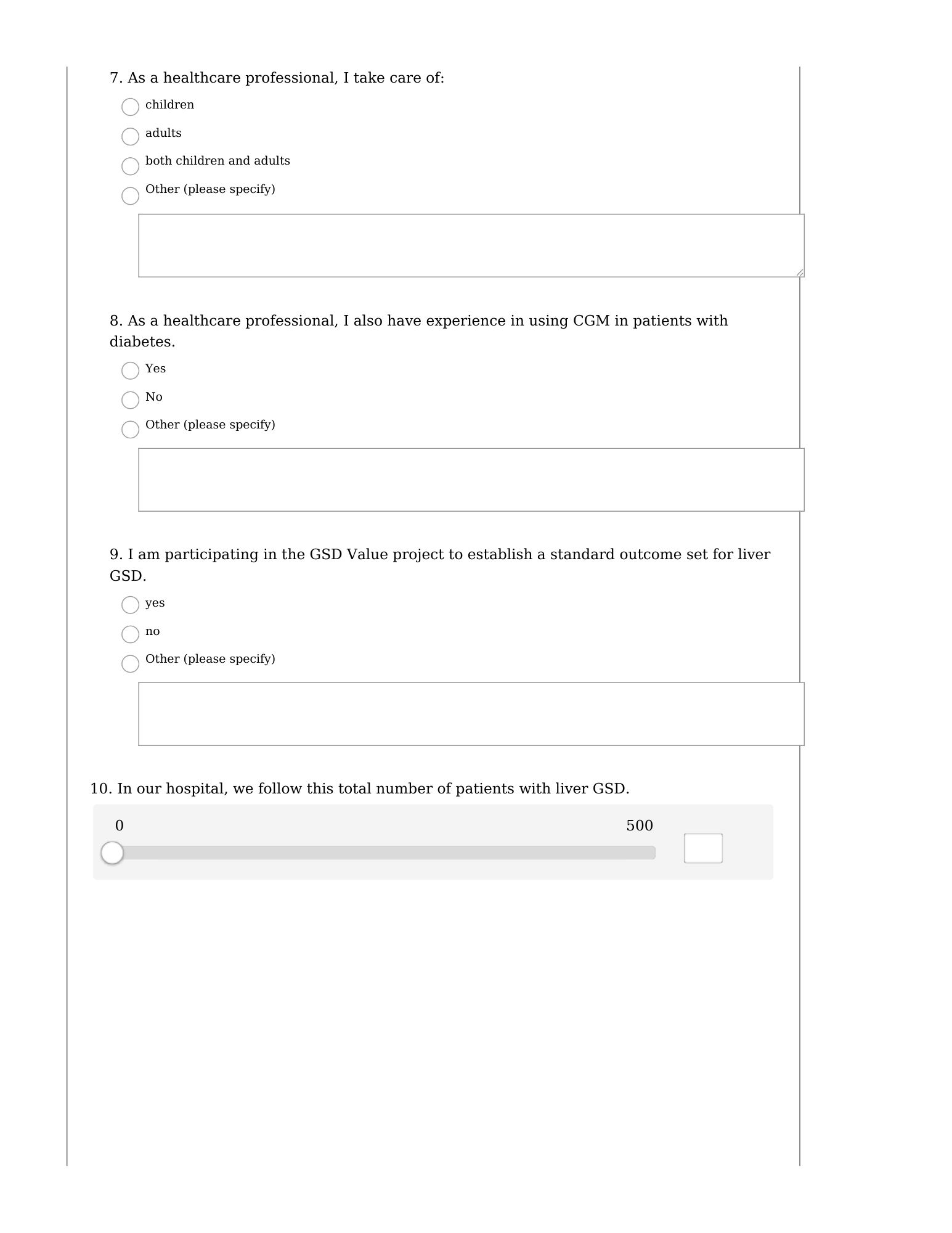

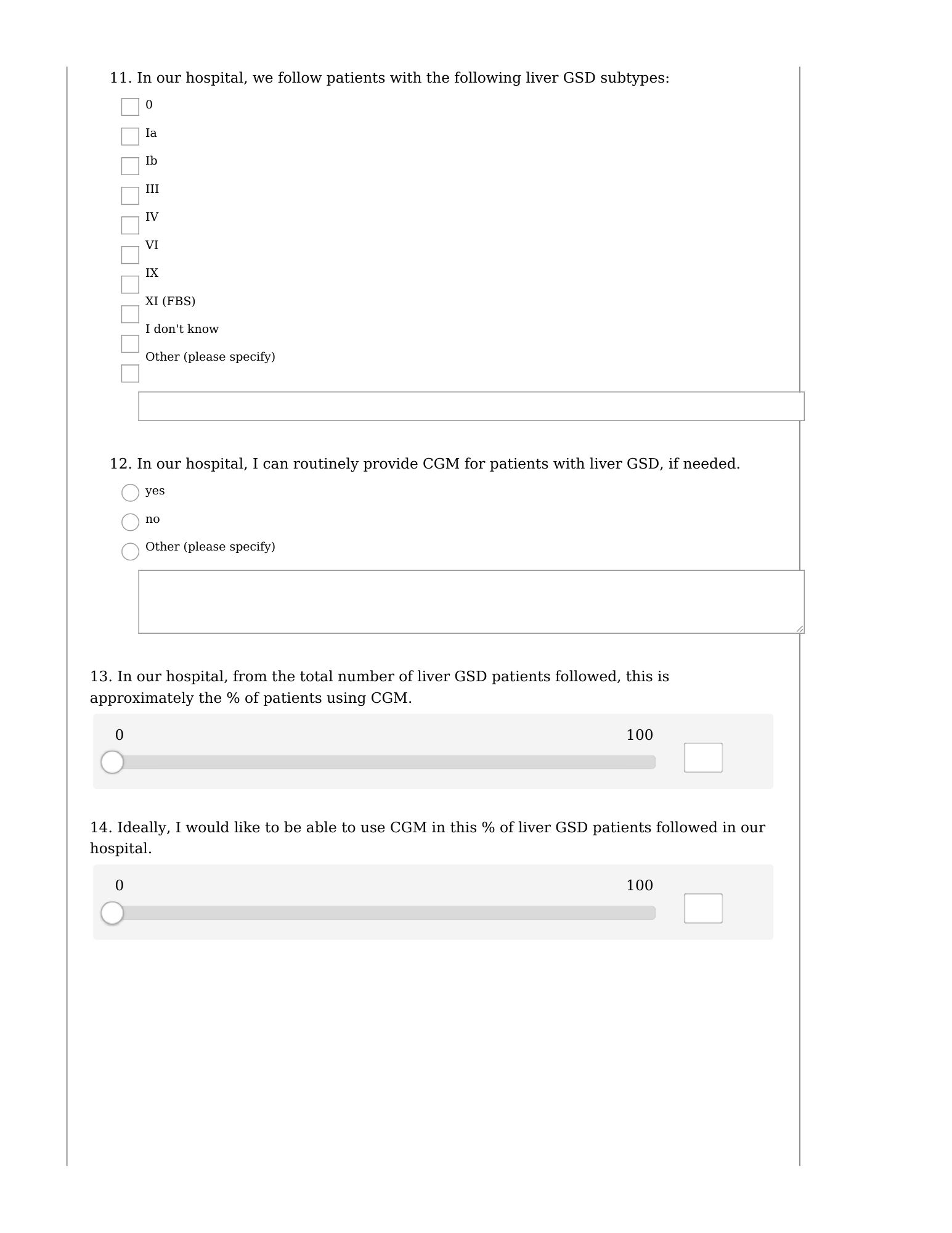

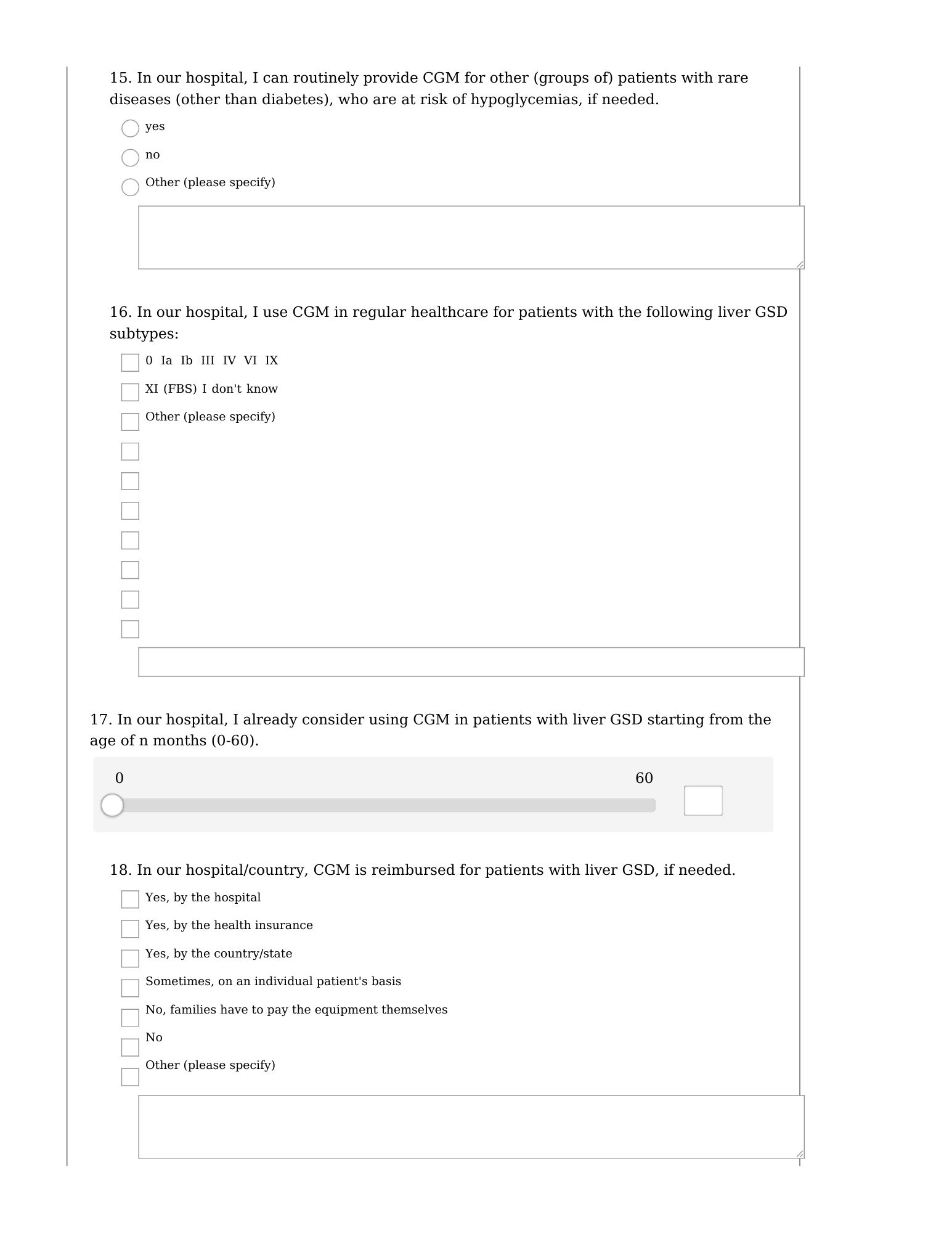

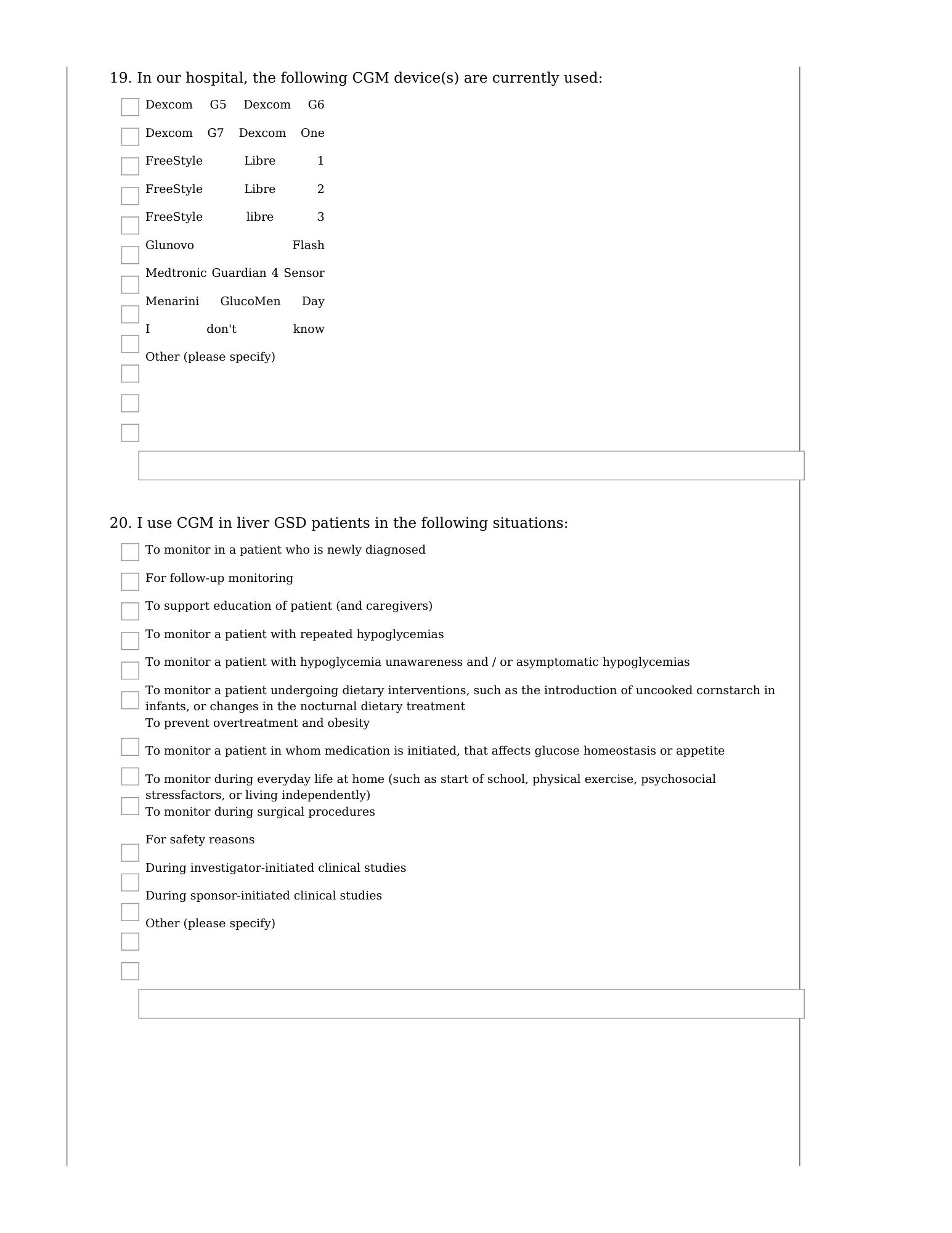

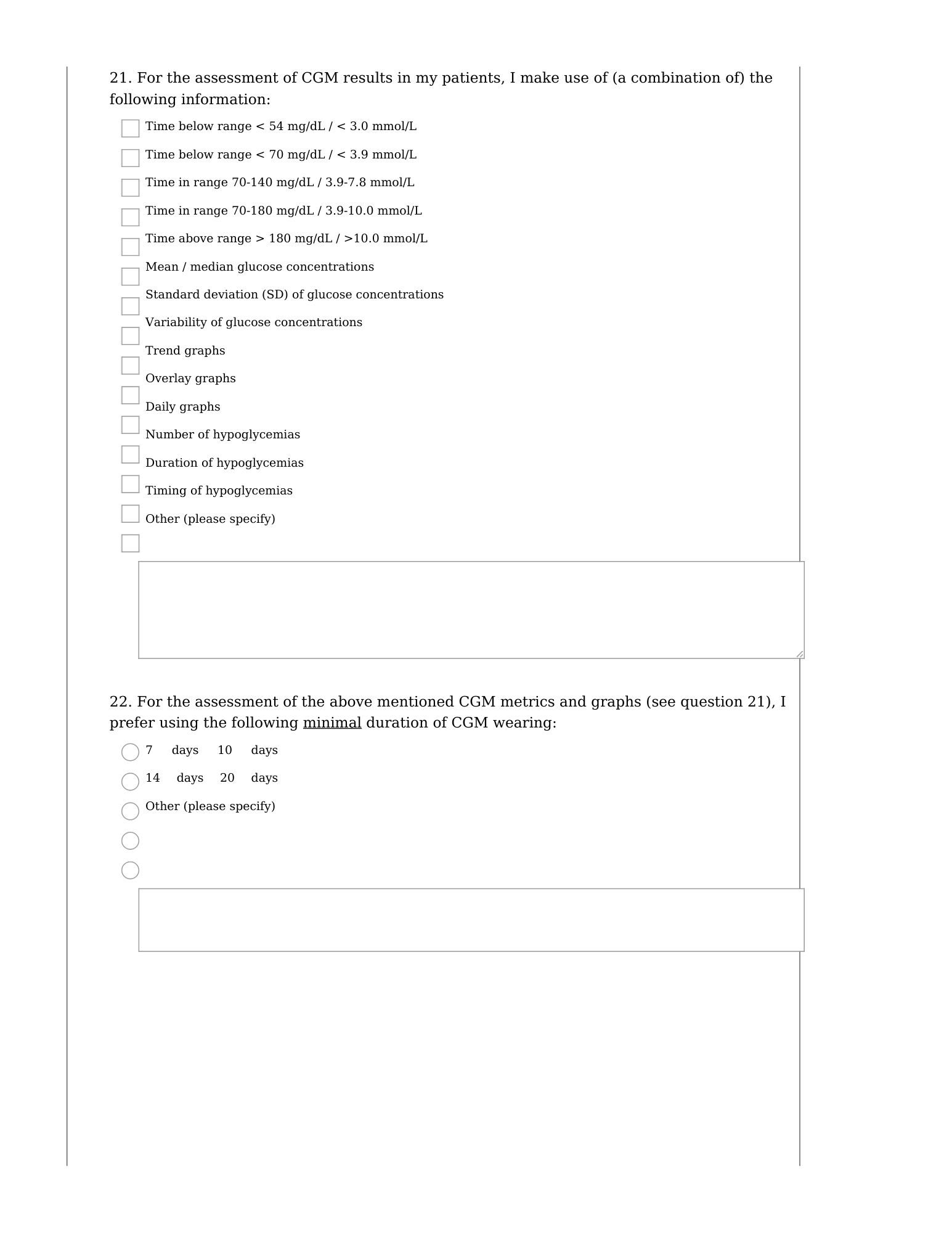

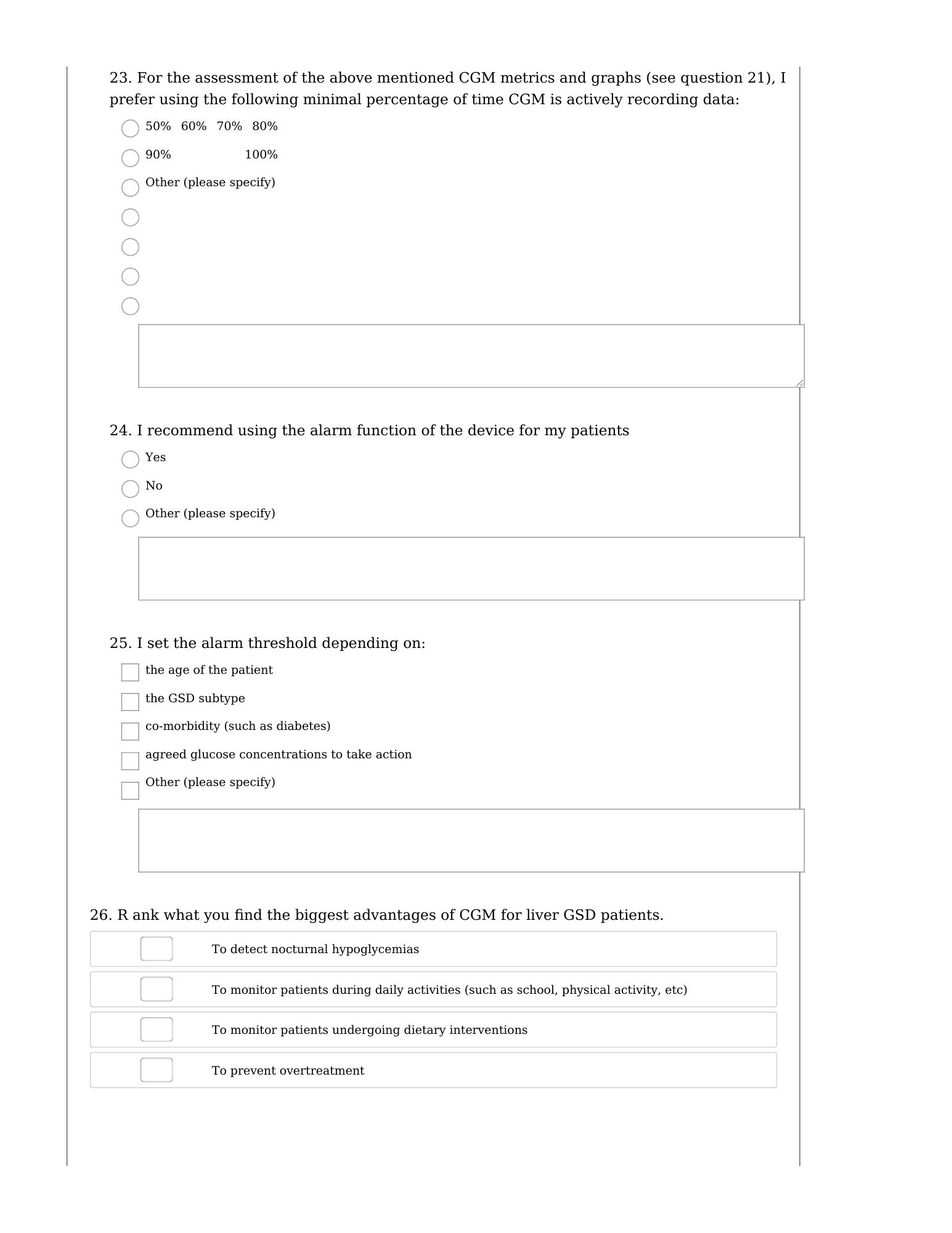

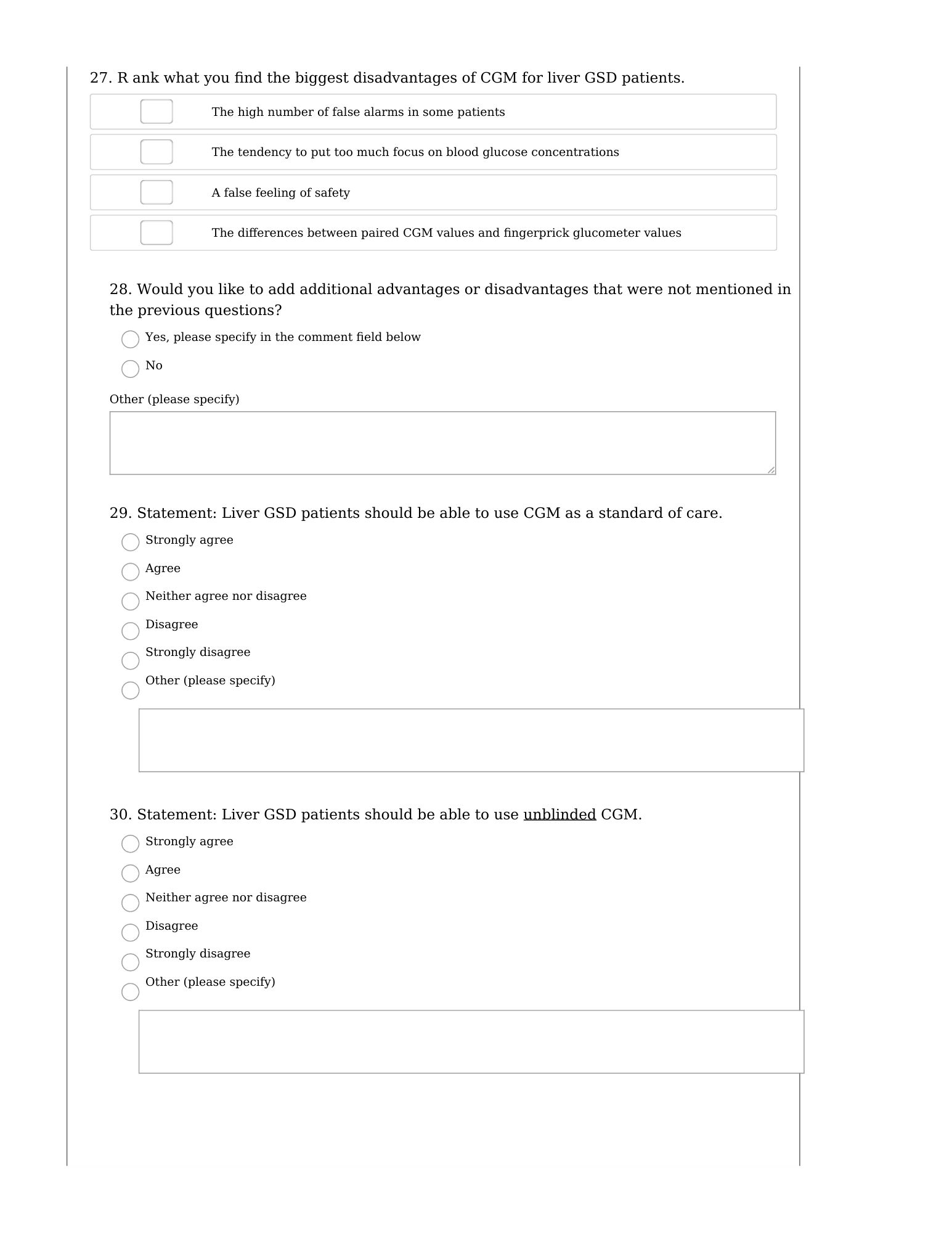

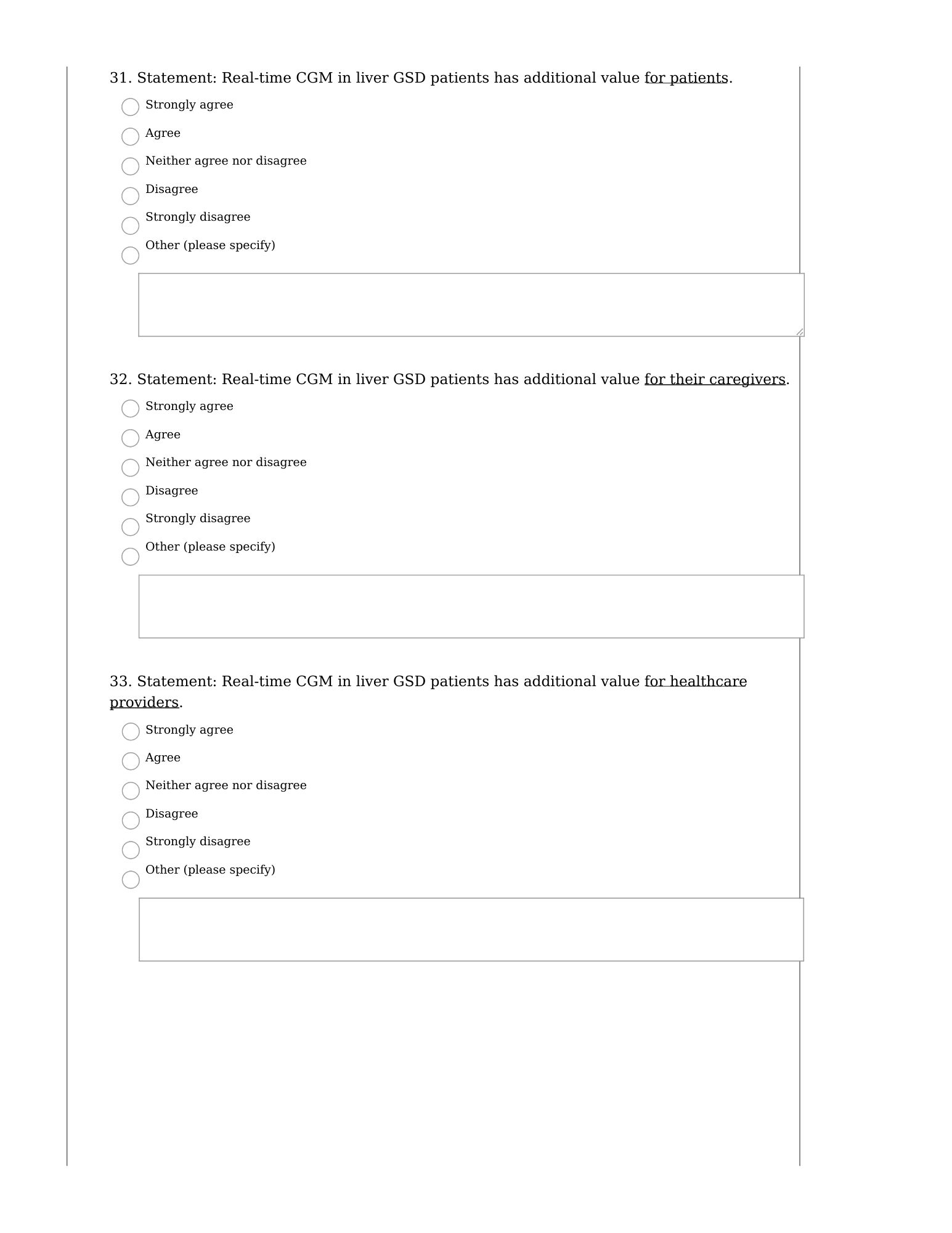

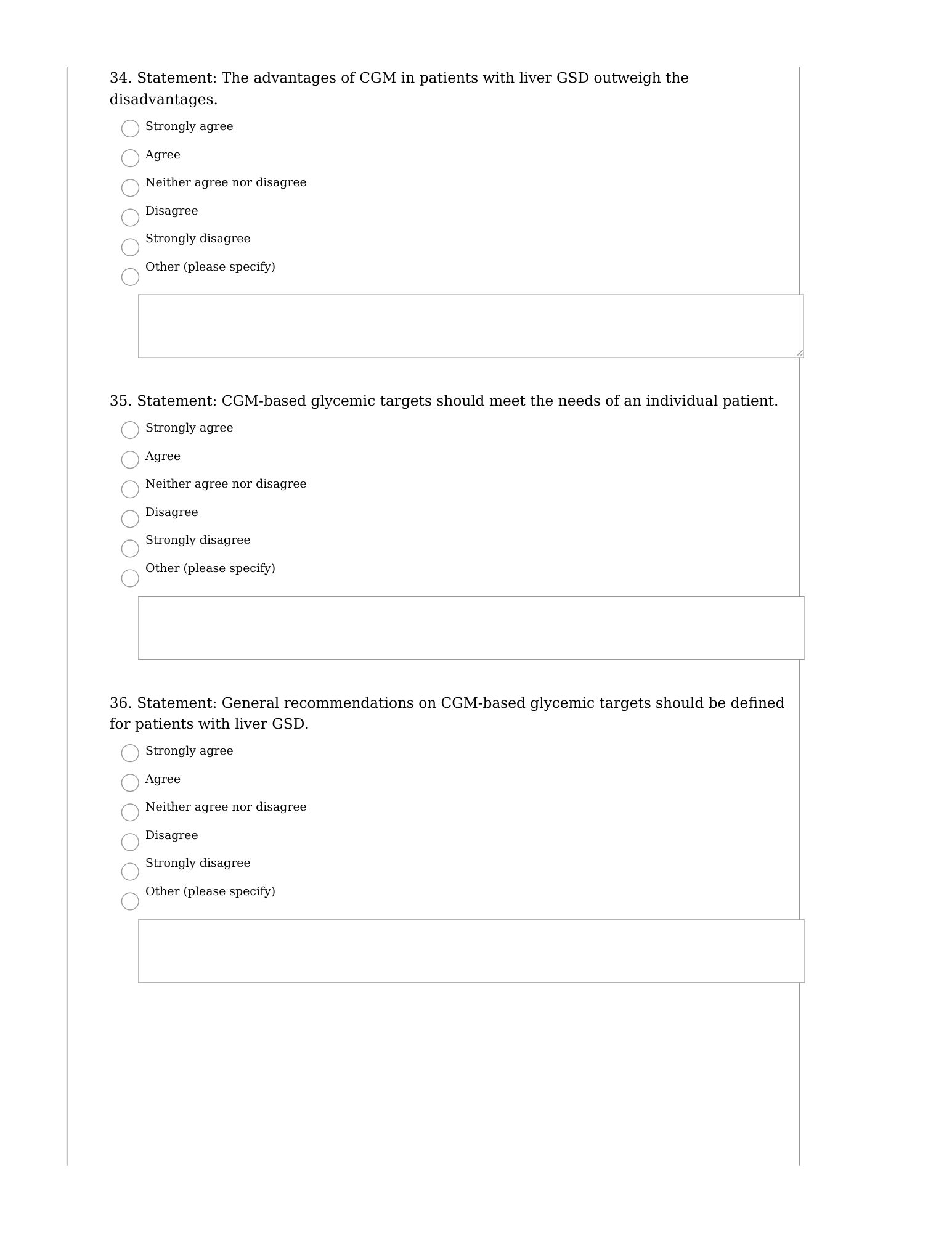

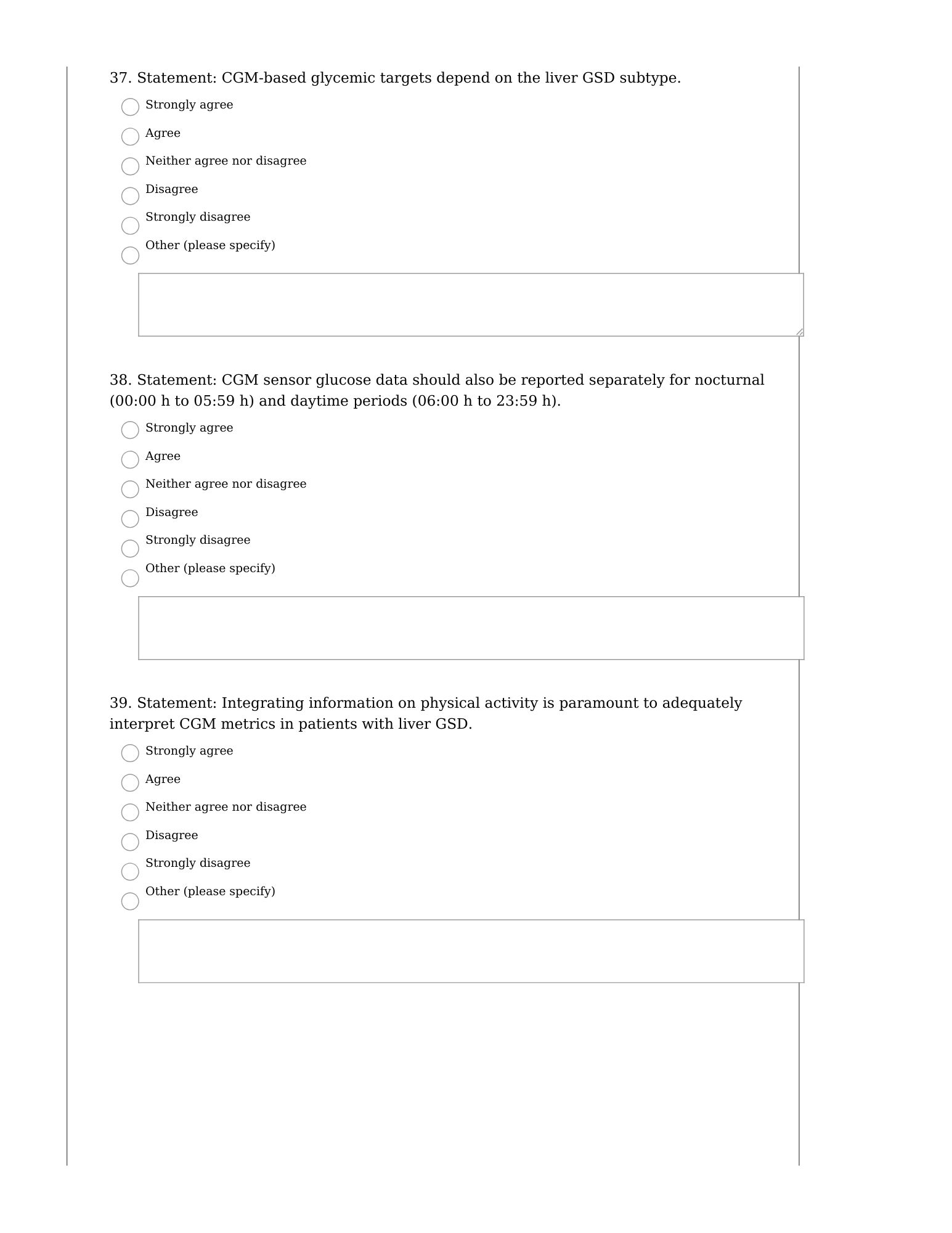

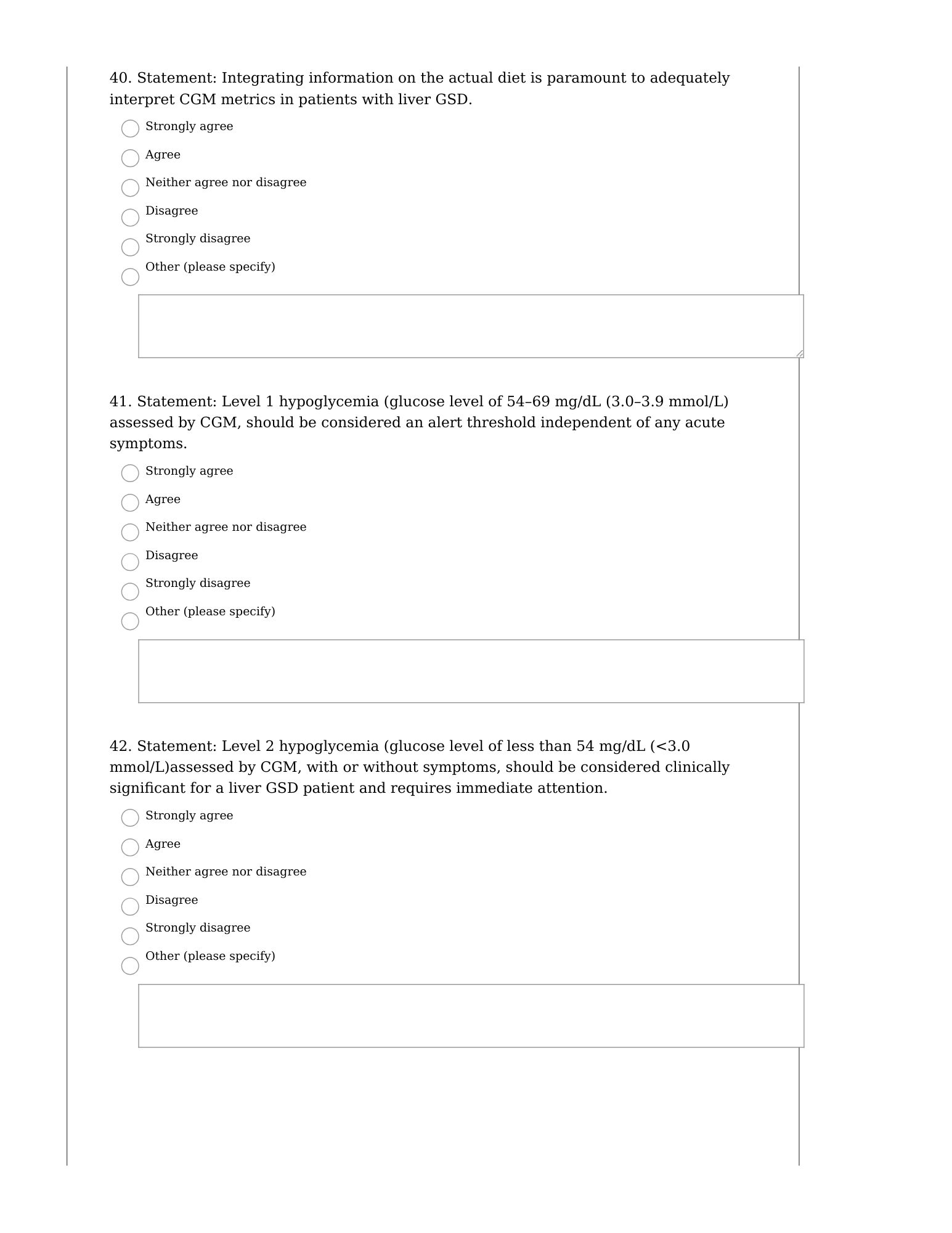

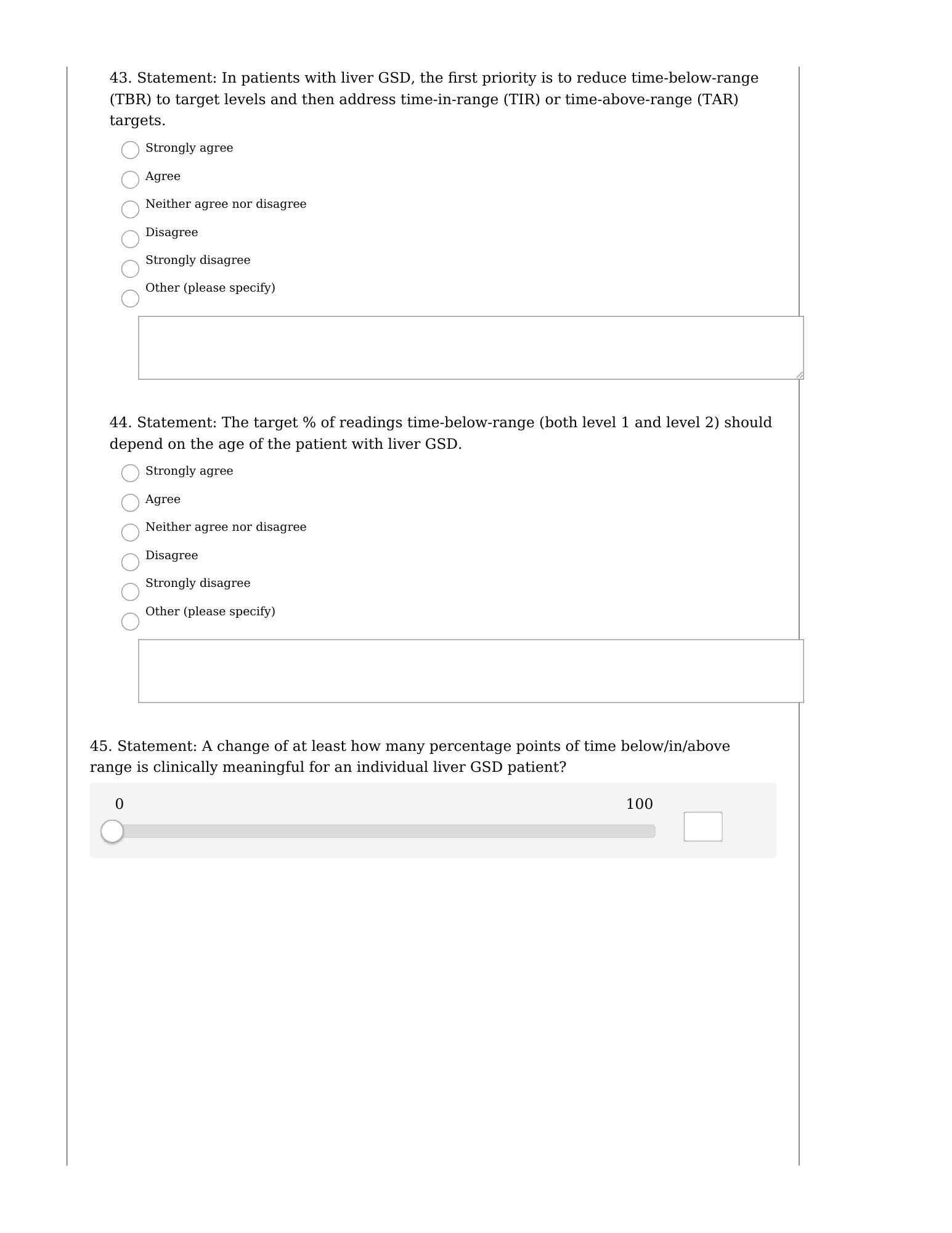

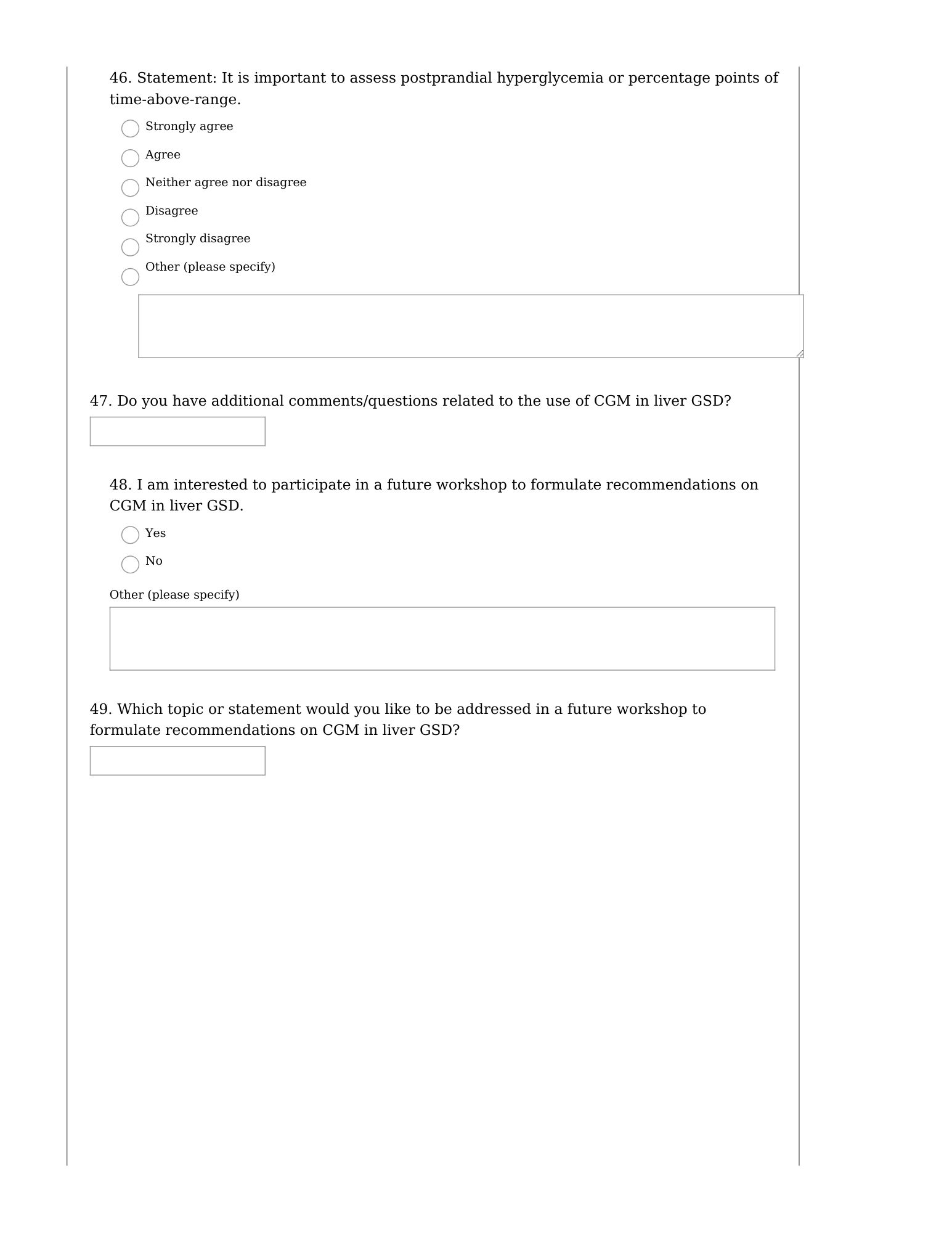


# **Supplementary File 2. SurveyMonkey® web-based questionnaire for people with liver GSD and caregivers (Q2).**

Please see the following pages for the printout version of the online survey.


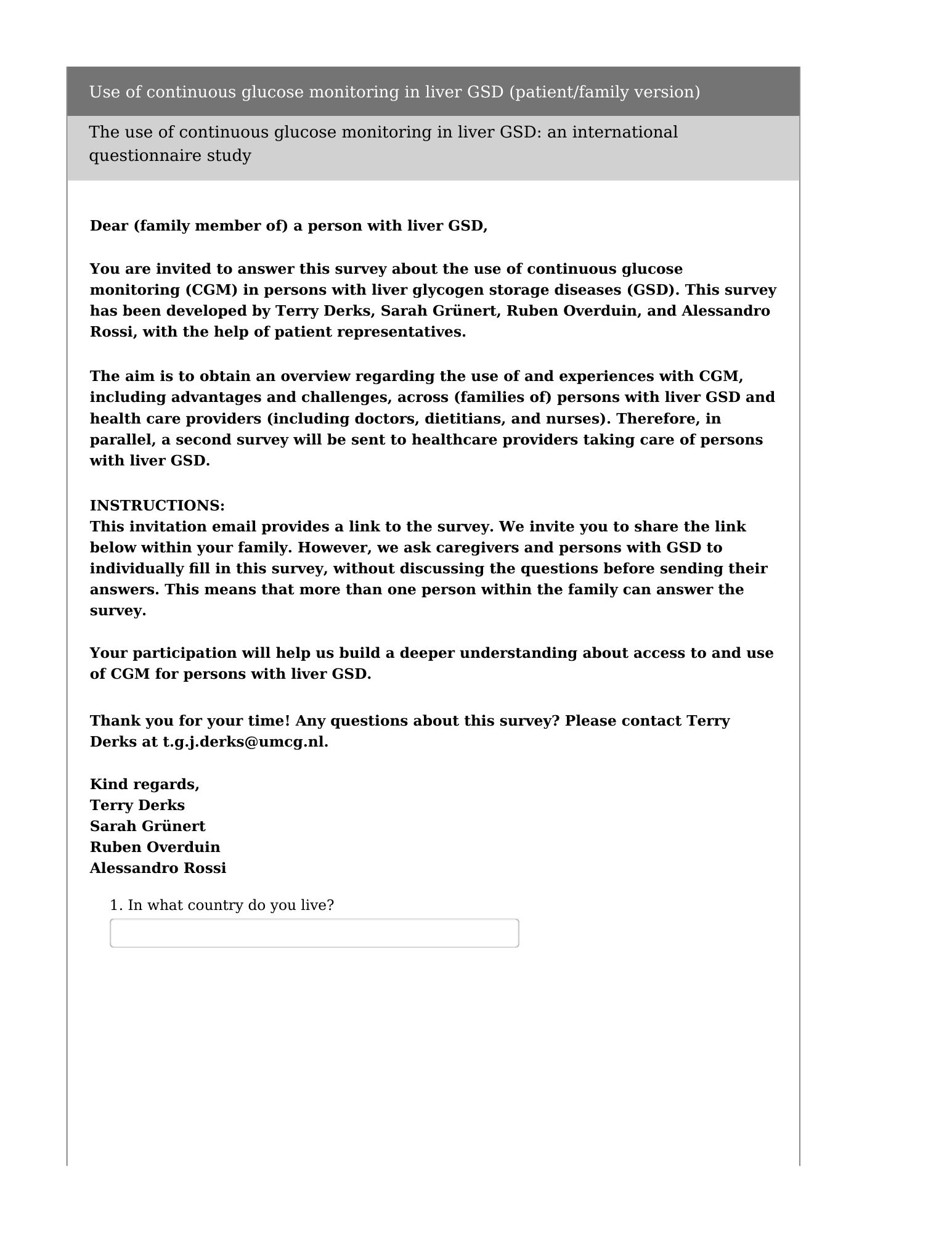

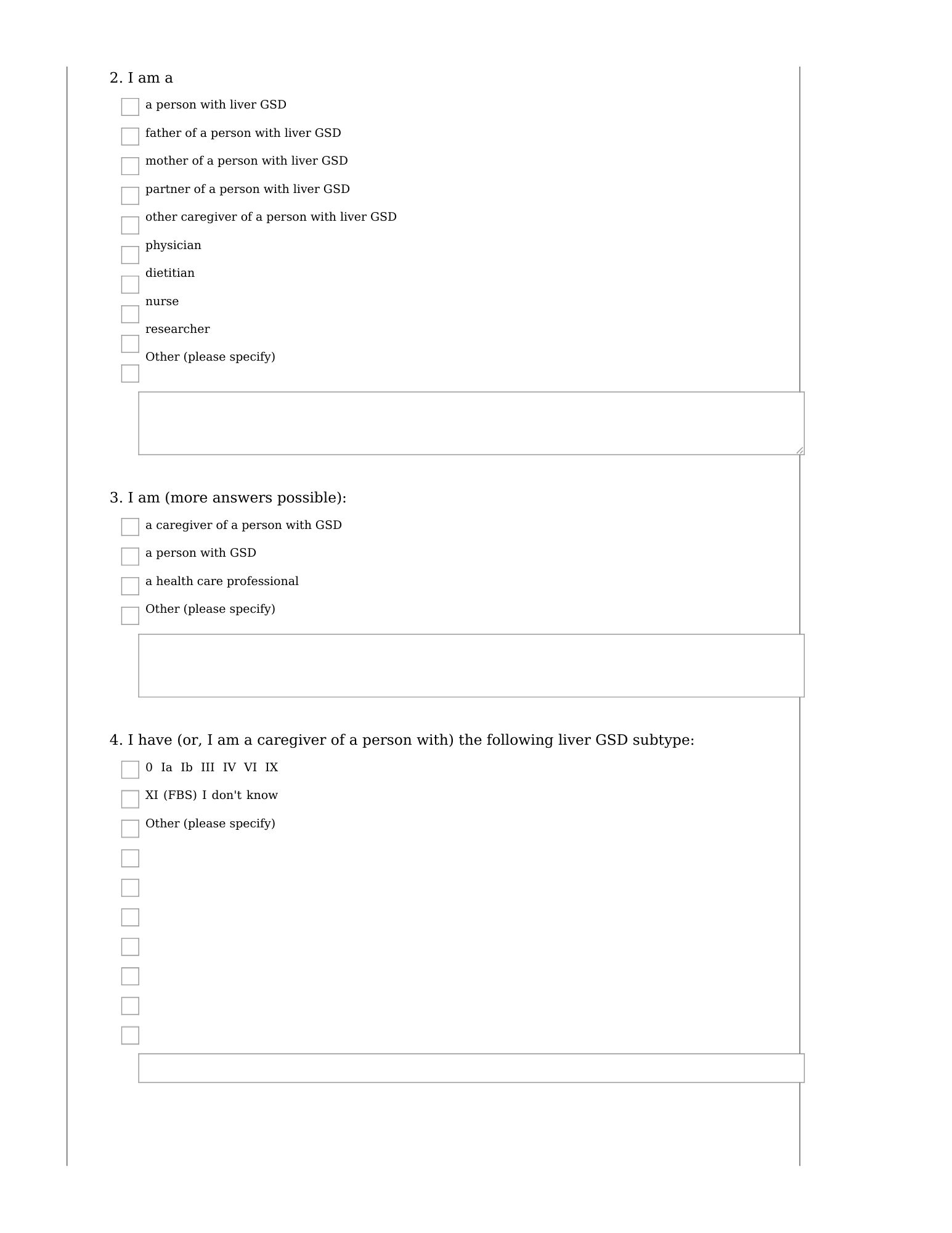

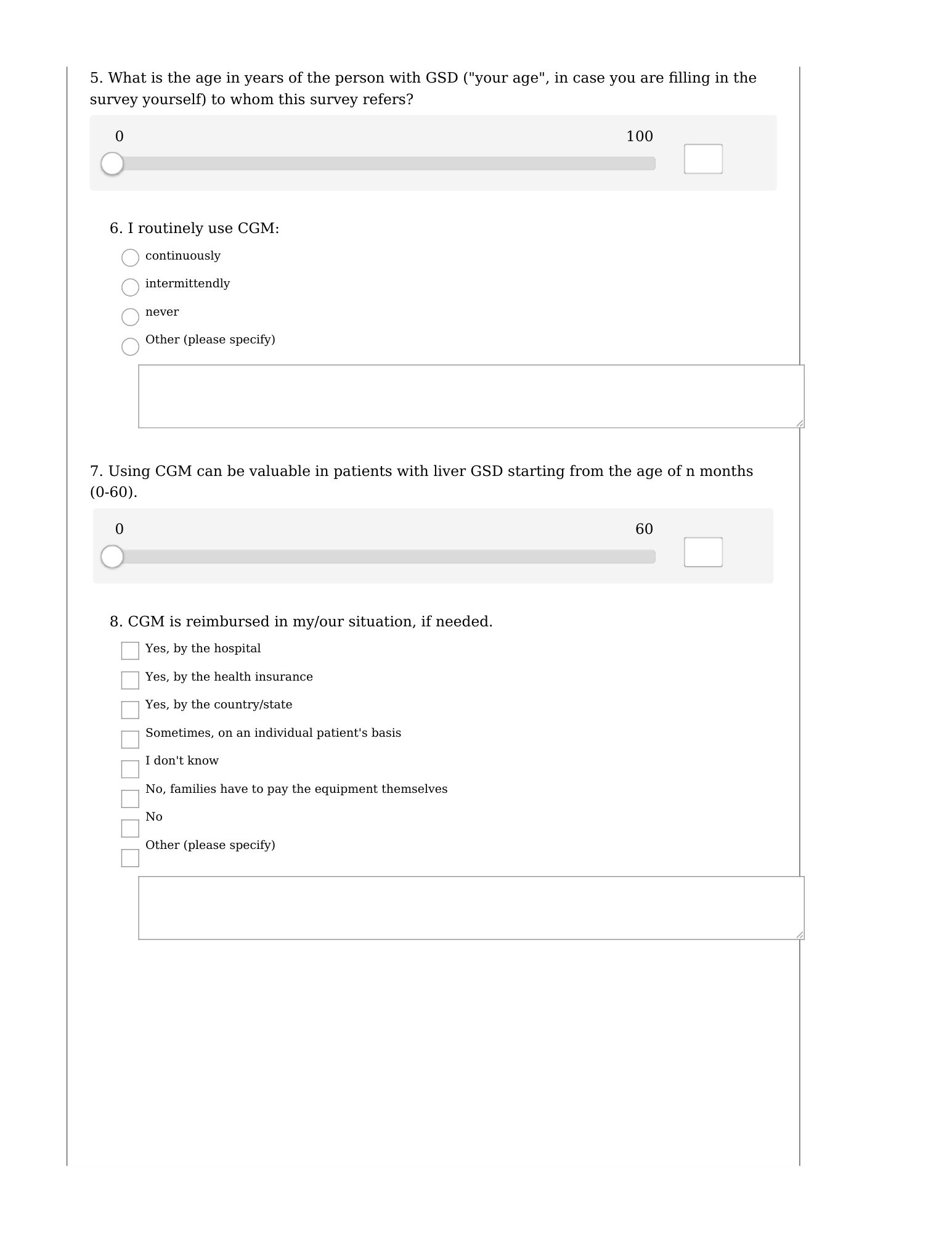

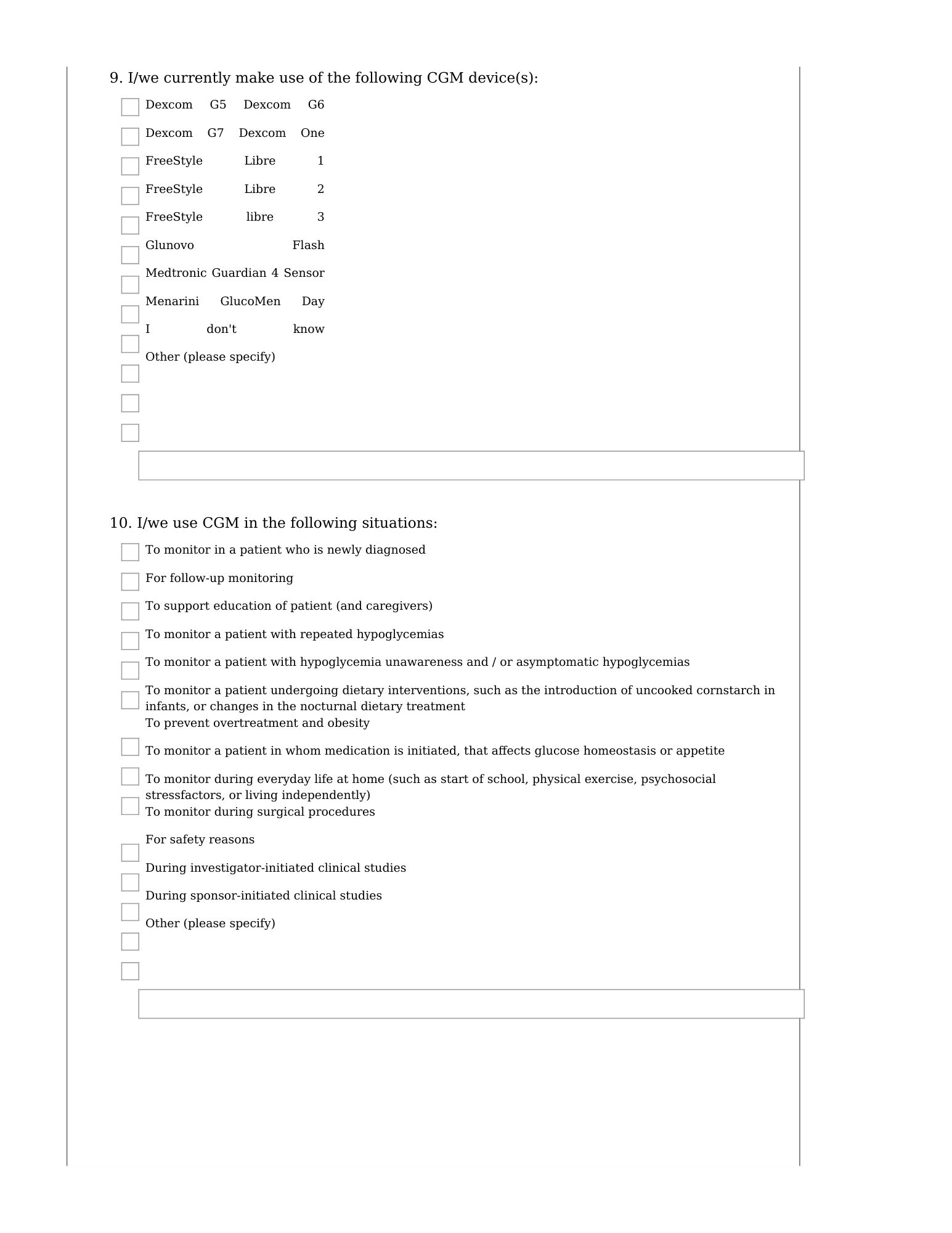

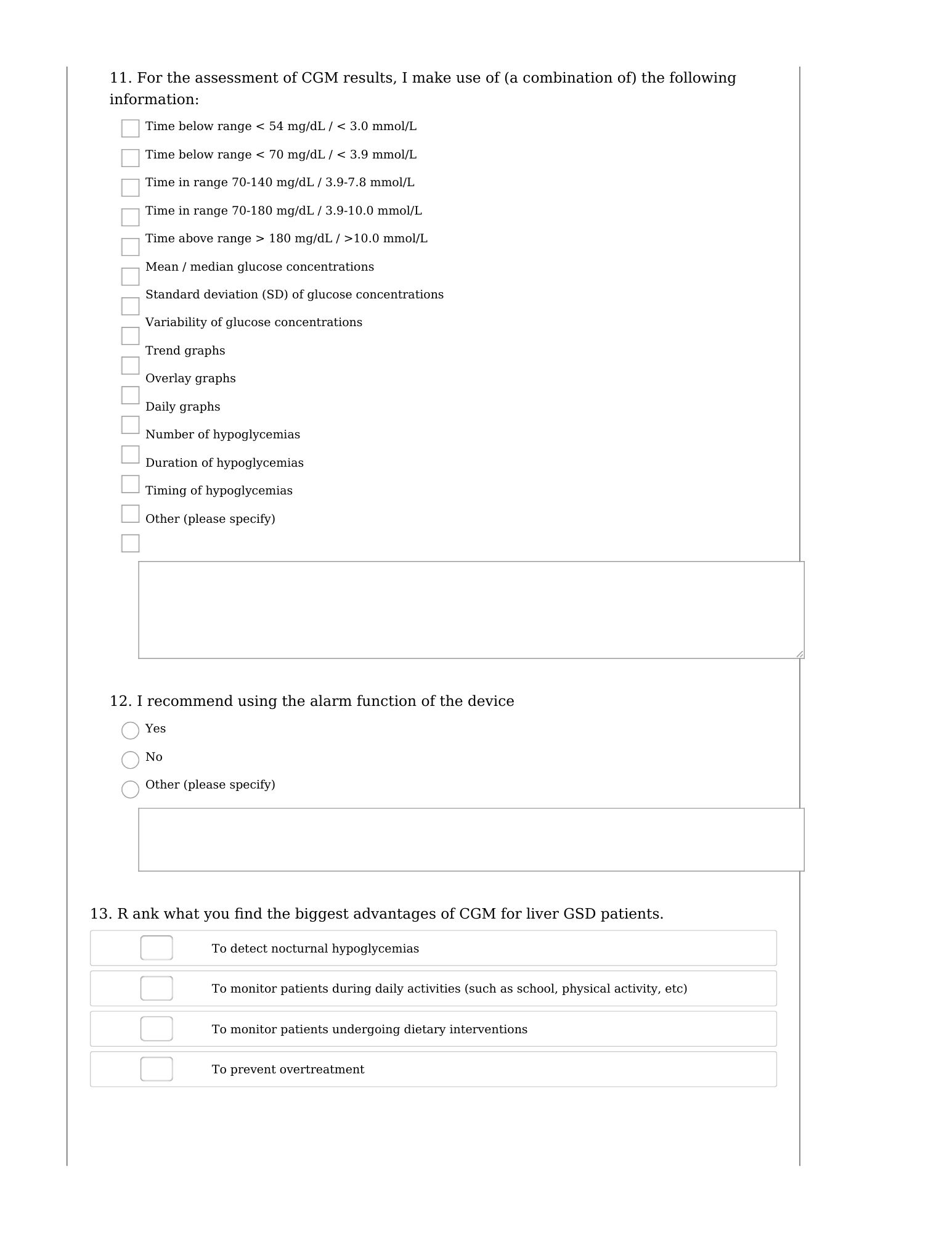

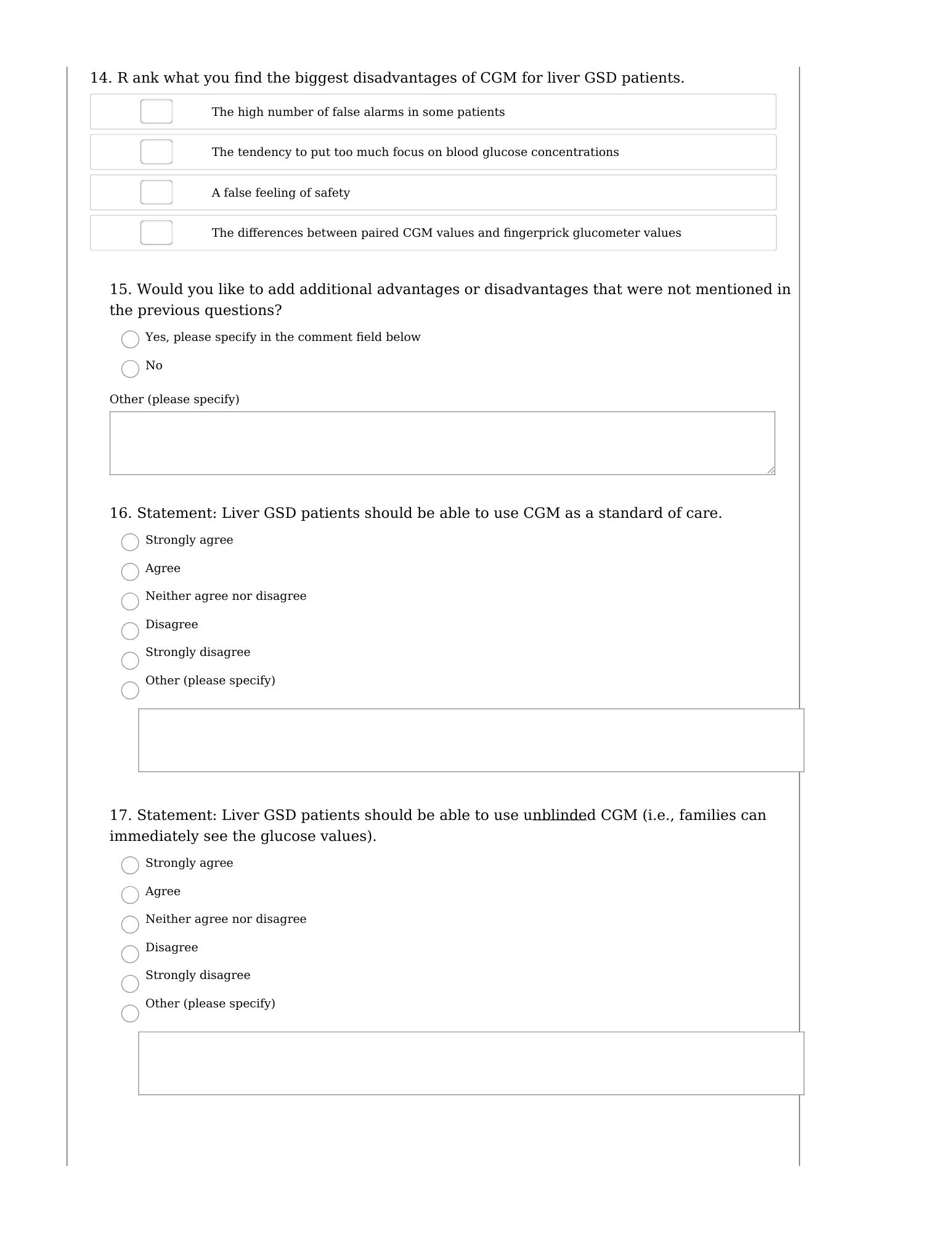

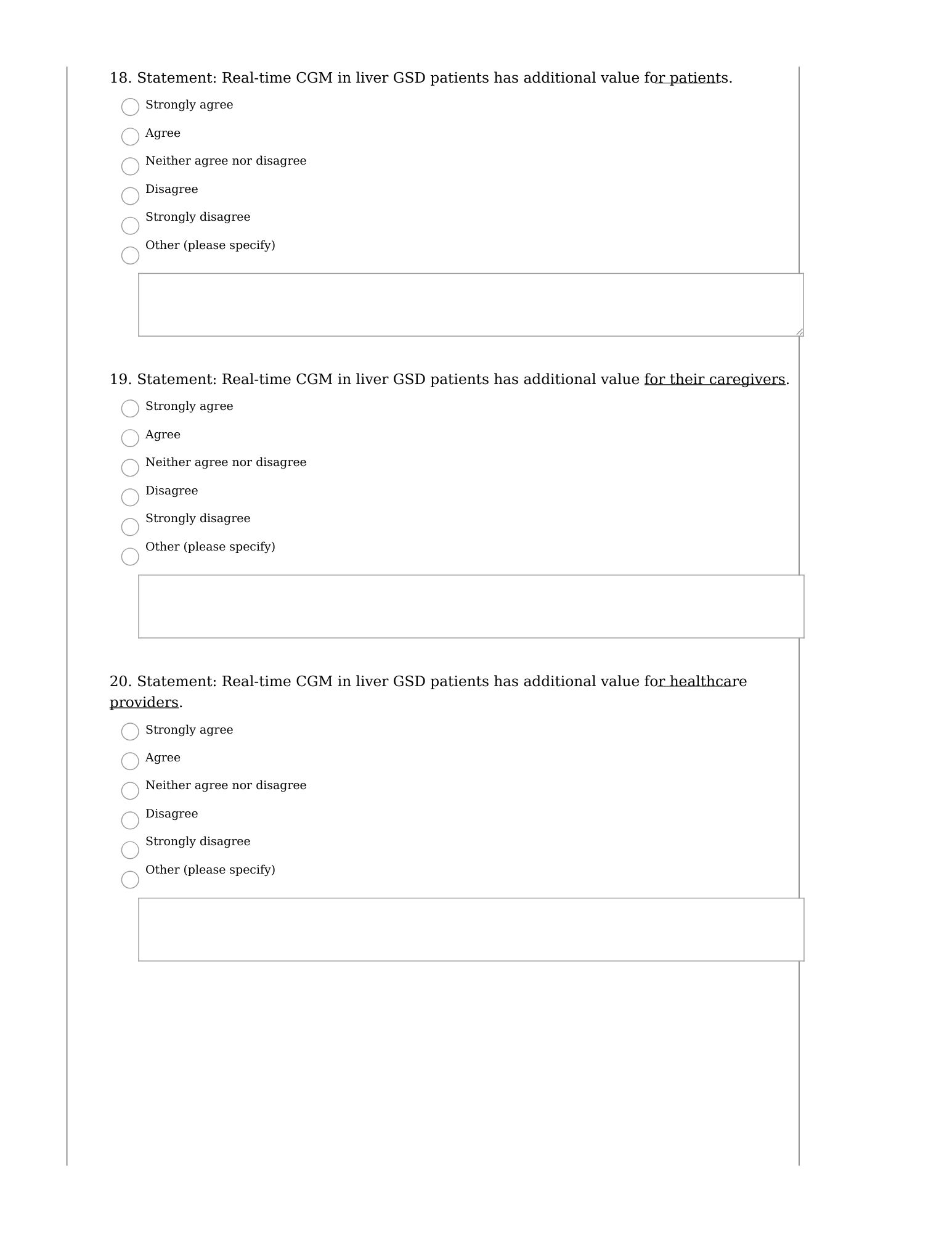

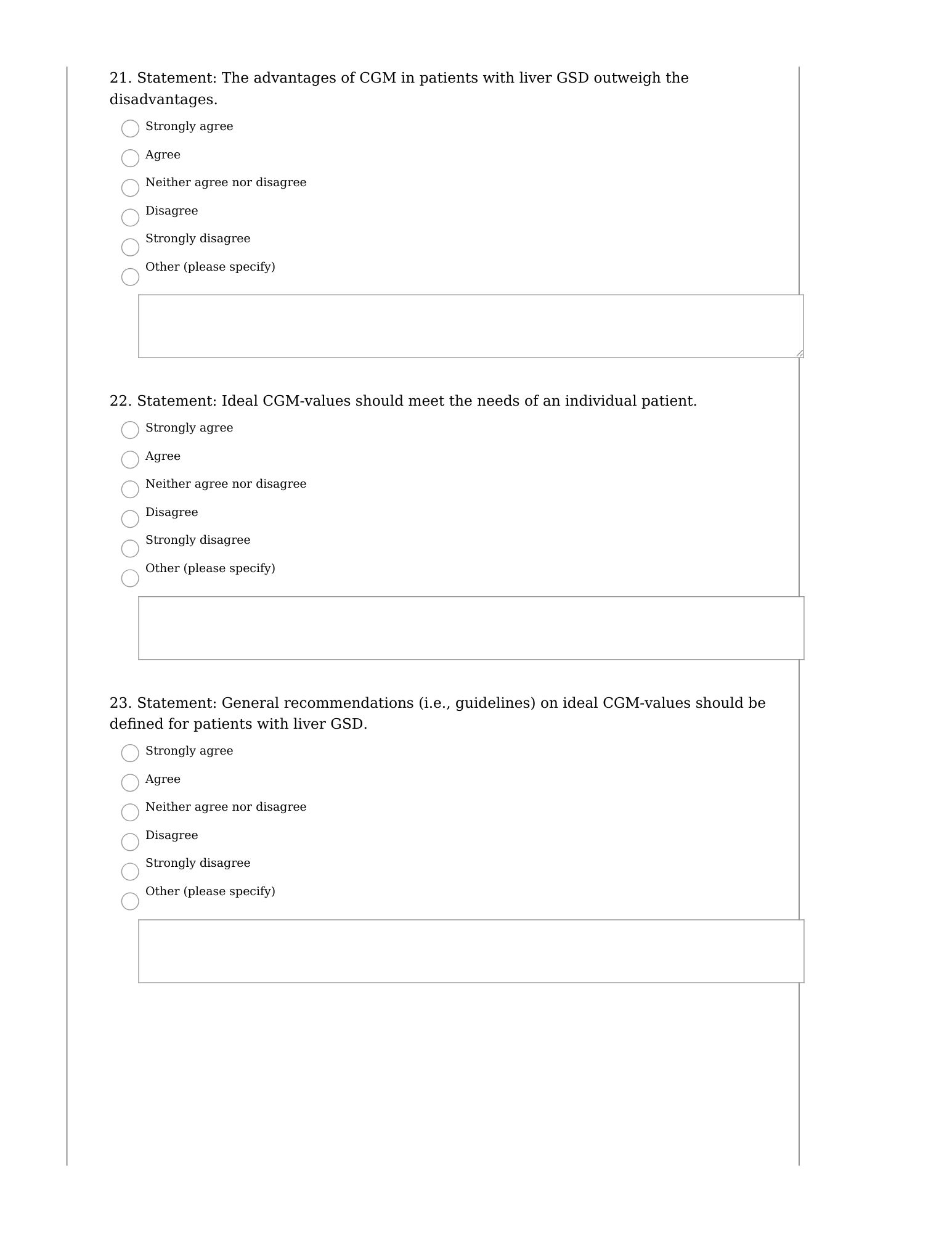

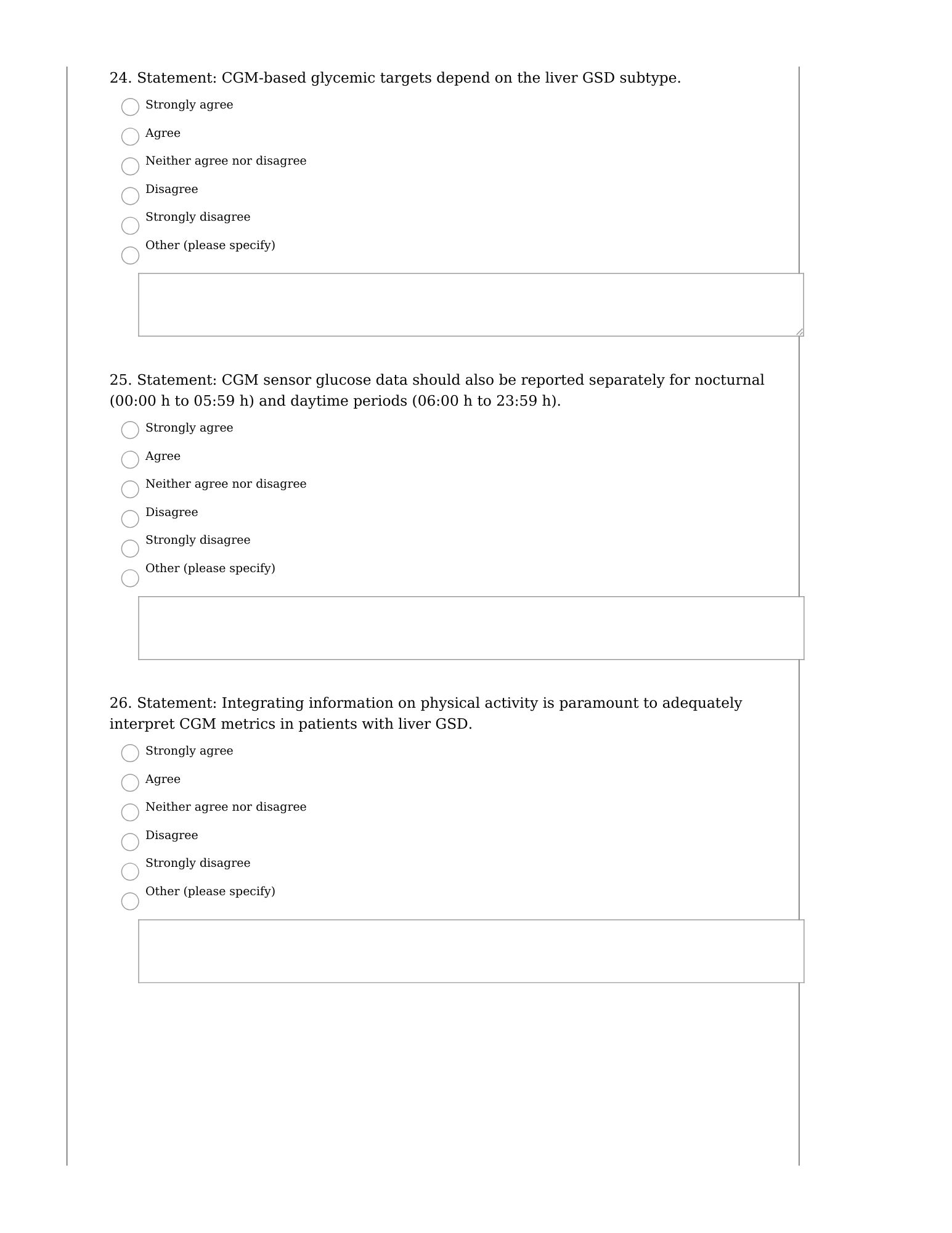

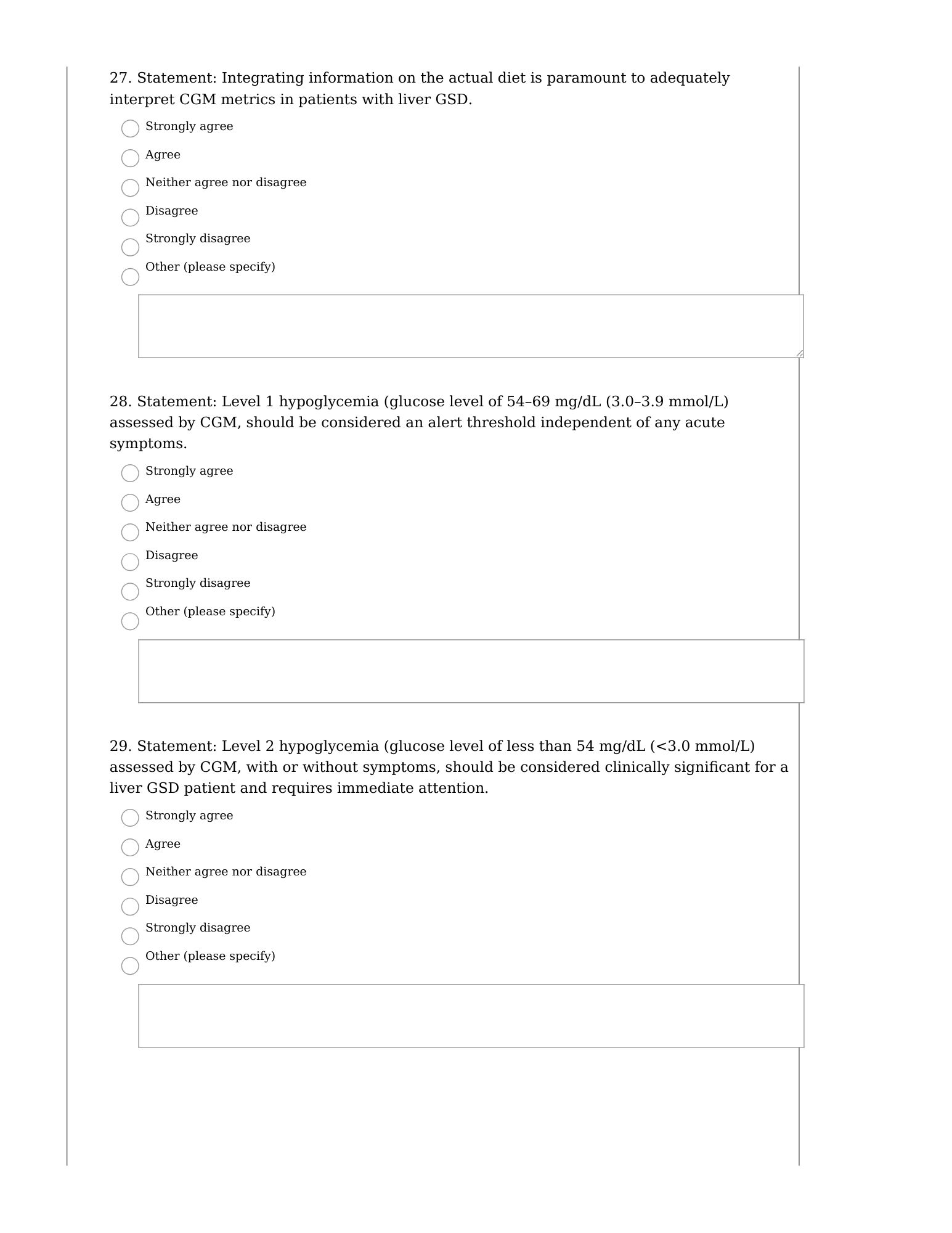

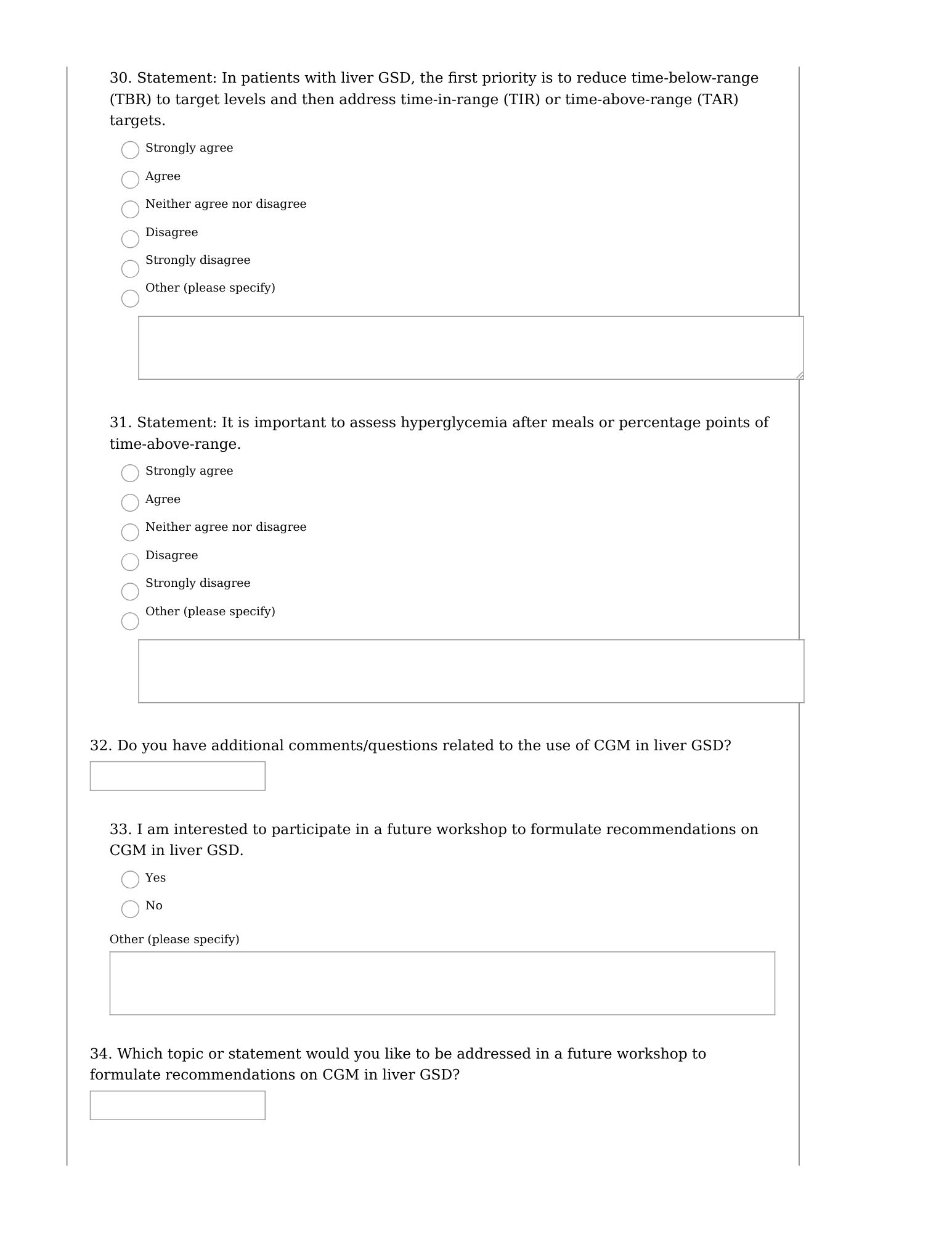


# **Supplementary File 3. Geographic distribution of questionnaire respondents.**

**
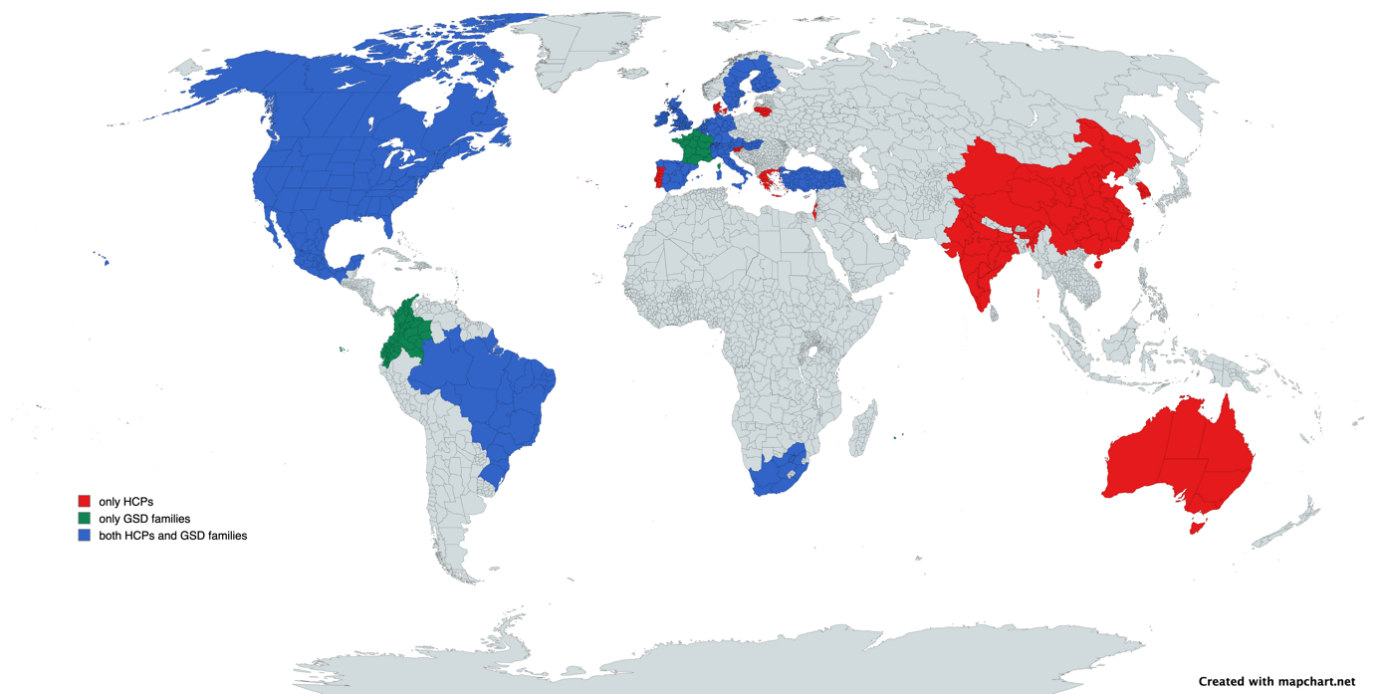
**

# **Supplementary File 4. Categorized advantages and disadvantages of using CGM in liver GSD according to HCPs (Q1, question 29; n=114) and GSD patients and caregivers (Q2, question 15; n=148).**

|  | **Advantages** | **Disadvantages** |
| --- | --- | --- |
| **HCPs** | *Impact on Daily Life*  Sometimes it is useful to monitor the blood sugars during sports (even retrospectively monitor trends)  Ability for more independence for older children and teens. Parents can monitor while patient is with friends or at school. Decreased anxiety related to fear of hypoglycemia.  Possibility to participate in daily activities such as kindergarten excursion, sports etc.  Advantage: followers and alarms on it  The school found this particularly helpful.  Fewer finger measurements (mentioned by two respondents)  *Increased Understanding of Therapy and Responses to Therapy*  More accurate reflection of daily life  Direct information on the glycemic effects of different foods.  Patients and caregivers can get an idea of the dynamics of blood glucose levels  Impact of not just dietary interventions, but also medication (e.g. empagliflozin for GSD1b)  Monitoring glucose continuously can clarify doubts of the family of the variations of glucose concentrations according to what they do at home with food, corn starch doses and the time of the dose.  Ability to trend glucose  Patients who wear a CGM often become more self-aware by their blood glucose and change the way they eat on their own, manage exercise including planning the timing of a shower so that their blood glucose can be maintained.  A CGM was very helpful in a family who was quite non-plussed by monitoring blood glucose. The measurements "woke them up" to what was actually going on.  CGM has revolutionized management of GSD in our center over the last 15 years. The information obtained is more meaningful than 24hr profiles in an inpatient setting. The prevention of over treatment is meaningful. | *Need for ancillary data*  It requires a detailed diary, which patients do not often provide.  Some patients do not put data into the hospital data base (Glooko). More complicated in older children that are more independent  dependency on hydration status of the patient; problematic use in infants (e.g. in hyperinsulinism)  *False Sense of Security*  Patients often experience neuroglycemia, which is not always reflected by Libre.  false negative results in case of hypoglycemia unawareness  In association with the false sense of safety, a decreased number of finger-sticks.  if healthy and stable fewer blood measurements with the risk of false measurements  *Accuracy/reliability*  Pressure lows that occur during night.  reliability of results  Accuracy  Delay in stabilizing blood glucose levels in the first few days and hyperestimated glycemic values compared to capillary glucose  The sensibility between a sensor and the next one. I found big differences. Big differences (more than normal) between paired CGM values and fingerprick glucometer values in pregnant women (sometimes more than 50 mg/dl).  sometimes CGM detect a hypoglycemia that does not correlate quite the capillary glucose, and patients with CGM tend to overtreat these hypoglycemias. Patients grow tired of the differences between CGM and fingerprick and abandon the CGM before they have learned to use it properly.  Effect of high blood ketones on CGM readings  Lack of correlation with lactate  false blood glucose level can be monitored in case of GSD patients with low blood sugar due to lactic acidosis.  poor correlation with lactate and ketone bodies (i.e. very indirect and unreliable measure of these) - thus, CGM needs to be complemented by further biochemical assessment  *Allergic Reactions (mentioned 5 times)*  Rarely, allergic reactions could be seen  some patients reacting to the glue of attached dressing - additional topical skin cover spray just sometimes prevents reactions  patient finds it itchy  Skin reactions to glue (redness, inflammation) can limit effectiveness.  *Technical Issues*  Difficulties in the calibration of the equipment also failures in the sensors confuse the patient.  Technical issues with the device cause problems.  Disadvantages: Sensor malfunctioning  Disadvantage: sensor total days they work is short.  Inaccuracy of CGM systems on days when it is placed and after several days of use.  *Anxiety*  some have tremendous anxiety about CGM use, can lead to some eating disorder behavior  Extra anxiety and burden in some cases  this device could provoke anxiety and put a lot of pressure on the patient and also, the patient may lose their ability to perceive the hypoglycemia by themself and become dependent on the technology.  Cost (mentioned three times)  Disadvantage: the CGM device is not affordable for the patients and my institution does not provide it. It is expensive  Disadvantage is cost  The cost remains a barrier for use of the product.  *Patient Preference*  Some patients just do not prefer wearing CGM devices; Costs  *Other*  loss of structure in the day because of depending on CGM when to eat |
| **GSD families** | *Improved Understanding of Disease:*  Adopt the correct diet avoiding hypoglycemia and hyperglycemia  Advantage: Improving the child's awareness about how hypo-/hyperglycemia feels  It helps you plan your day and ensure that you don't forget to eat (especially in stressful situations)  We are feeling very comfortable to use Freestyle Libre 3 for our son who is 5 years old. It is so good to see how food is influencing his blood sugar and that we are showing him that he is aware. We would give him much faster Maltodextrin, if we do not would see how long different food is holding blood sugar. We are so pleased that this type of monitoring is available and invented.  Know when to eat, if I forgot or if it lasts longer than calculated  With the CGM you can achieve better blood sugar control so that organ damage can be avoided.  *Safety:*  With continuous feeding at night (very common in France) it’s for me a life saver and it HAS to be put with overnight continuous feeding.  We have found it very useful to use continuously and it has been lifesaving with overnight hypoglycemia  Good when unwell and sometimes blood glucose levels get lower more quickly than usual.  Very helpful to monitor during sports / sickness when calculated portions are not working.  Allowing others follow glucose concentration levels as a backup for safety reasons. Missed cornstarch doses can be caught especially if GSD person sleep through a cornstarch dose.  Biggest advantage question 13 is monitoring during sickness at number 1  Low blood sugar alarm function  To detect any kind of hypoglycemia not only nocturnal.  To have several followers which can provide help.  *Independence:*  Our daughter has an intellectual delay this helps us to give her some independence.  It’s easier to let the child [go with] to someone else.  Helps to monitor sports activities and improve intake (advantage)  I think it’s very beneficial for patient independence being able to share numbers live with parents/significant other.  The ability of caregivers to monitor older children/teens remotely-- the patient gets more freedom, caregivers get more peace of mind. Remote monitoring. It helps us monitor her at school and playing sports as well. It gives caregiver some relaxation to.  *Less invasive:*  …And there are less pokes as well  If working properly, is a great advantage because it saves a lot of punctures, especially in small children  Tracking blood sugar from other place, not often measuring with fingerprick  That I don't have to fingerprick my finger  *General:*  There are no disadvantages. It's just important that families learn how to properly use the continuous CGM and the information it provides.  Patients need to understand the limitations and how the graph smoothing works. Having families say to other families that the tool is inaccurate or to ignore for 24 hours is unhelpful and potentially harmful.  I think it’s a wonderful device when it is working correctly.  Continuous blood glucose monitoring | *Allergies/Discomfort*  Allergic skin reaction around the sensor  The sensor sting hurts. My daughters have an allergic reaction to the sensor's adhesive.  Have to change it every 10 days  Hurts sometimes when I placed it on a bad area  Allergic reaction to the "glue" used for the patch.  Change every 14 days.  Rash in the arm.  Disadvantage: Irritation of the skin.  My son does not like having it on because it irritates his skin  It is a pain to change captor every 10 days  Wound healing on the arms  *Does Not Replace Usual Monitoring:*  We use the CGM, but still finger stick every few hours or to verify a low. It is a very helpful tool, but for us it does not replace traditional finger sticks.  *Inherent Problems:*  CGM is designed for diabetes patients. The target area (graphical representation) cannot be adapted to the needs of GSD-Patients (e.g. 80-120). In this way the self-monitoring is assessed too positively (time in range etc.), because hypoglycemias usually take a relatively short time-period, but are a critical event. We need a much higher quality of CGM values! The differences to the real fingerpick glucometer values are often enough too big. This well-known differences makes too much troubles and stress: When CGM is running good, then it is really helpful and stressless. But when CGM is running bad, then it is an additional psycho-horror with a bad body-feeling by hypoglycemias and not helpful. CGM values are not safe enough in real life. I once used the Abbott freestyle brand glucometer sensor but I was not sure when I had hypoglycemia since when it was taken in blood the values were very different. It’s too slow. I have hypoglycemia within 5-10 Minutes. I feel sick, but CGM shows good values. In blood the hypo is clear. After 10-15 minutes the CGM shows the values too but it’s only good for daily profile of blood sugar in my opinion. CGM Dexcom is made for diabetic, you can only choose in ranking between >300 mg or >400mg/dl, so the graphic is not as detailed AS WE needed. Often the values ​​cannot be traced back to the capillary stick (lack of correlation)Lack of calibration option on Libre 3  *Delays in Monitoring:*  Delay in monitoring glucose levels (disadvantage) -> does not always help to prevent hypo's. The sensor cannot keep up with the rapid fluctuations in BG on Type 1a. When fitted it didn't show true low blood sugar readings they were originally catered for diabetics. When the new sensor is inserted, it takes up to 24 hours for the value from the sensor and the value of the blood-measured sugar to adapt to one another. The fluctuation range at the beginning of the sensor being set is very high. Time of uncertainty after newly placed CGM because values are not correct for up to 48h  *Incomplete Monitoring;*  I rather would have a combination of a meter who measure glucose and ketones at the same time in our case the glucose aren’t that low any more but ketones give us more information. And of course always look at clinical appearance of the person with low blood sugar instead of measurements. Disadvantage: Does not cover ketone monitoring yet (for ketotic types)  *False Values:*  In some cases, especially when the transmitter’s battery life is towards end gives irrelevant values, false alarms and huge difference between finger prick and CGM receiver. Too many false values. Too many false measurement results  *Alarms:*  Disadvantage: alarm while eating (you cannot pause for 15 minutes on Freestyle Libre 3)  some false alarms  no real time  No possibility of lowering the alarm sound according to the phone settings.  Frequent alarms can be stressful.  Would like “followers” to get notifications regarding when Dexcom needs to be changed.  *Use in Children:*  use of CGM sensor for little children is a challenge, especially the application at the proposed location on the body (we started with Freestyle 2 on our 2yrs old child).  They are not reliable for infants  *Equipment Failures:*  Furthermore, the failure of the CGM (falls off or sensor reports failure and need of replacement)  Defect before expiry of the expected lifetime  Unreliability of the equipment that cannot last 2 weeks  Another disadvantage is the sensor can become faulty. It has happened numerous times to my dad (diabetic) who also uses a CGM.  *Placement*:  Inaccuracy due to lying on it at night or during sitting  Inaccuracy when placed at a bad area  Don’t know what areas are bad before placement  The pressure sensitivity of the sensor during sleep, then the values are usually lower  *Cost*:  I can't use it permanently because it's not paid for by insurance. Only diabetes patients have this opportunity.  High cost /month  *False Sense of Security:*  Parents only rely on the monitor and no longer notice symptoms of hypoglycemia in their child. They measure every half hour at night and a child no longer has the chance to get to know their body and the symptoms themselves.  Tendency to neglect ketone testing (disadvantage) |
